# Marine Natural Products in Preclinical Cancer Studies: Ten Years of Advanced Total Synthesis

**DOI:** 10.3390/md23110430

**Published:** 2025-11-07

**Authors:** Ester Colarusso, Assunta Giordano, Maria Giovanna Chini, Giuseppe Bifulco, Gianluigi Lauro

**Affiliations:** 1 Department of Pharmacy, University of Salerno, Via Giovanni Paolo II 132, 84084 Fisciano, SA, Italy; ecolarusso@unisa.it; 2 Institute of Biomolecular Chemistry (ICB), Consiglio Nazionale Delle Ricerche (CNR), Via Campi Flegrei 34, 80078 Pozzuoli, NA, Italy; assunta.giordano@cnr.it; 3 Department of Biosciences and Territory, University of Molise, Contrada Fonte Lappone, 86090 Pesche, IS, Italy; mariagiovanna.chini@unimol.it

**Keywords:** marine-derived compounds, anticancer activities, total synthesis

## Abstract

Marine ecosystems represent an exceptional reservoir of structurally diverse metabolites with remarkable pharmacological potential. Over the past decades, the exploration of marine organisms has led to the discovery of an ever-expanding number of bioactive compounds. Many of these metabolites display highly original chemical scaffolds that are not typically found in terrestrial organisms, offering new opportunities for drug discovery. Among the most promising applications is their development as anticancer agents, given their ability to interfere with key cellular processes. This review highlights marine natural products currently under investigation in preclinical studies as potential anticancer lead compounds. The molecules are classified into major structural families: aromatic and heterocyclic alkaloids, terpenes and their derivatives, macrolide frameworks, and diverse peptide-based scaffolds, alongside other complex classes (polyketides, thiazole lipids, alkylamino alcohols, and pyrrolocarbazole derivatives). A particular emphasis has been placed on the role of total synthesis over the last decade. Advances in synthetic methodology have not only enabled the production of these complex metabolites in sufficient quantities but have also facilitated the development of novel chemotherapeutic agents. To overcome the challenges of limited natural availability, the advanced synthetic approaches are crucial for harnessing the full therapeutic potential of marine-derived compounds.

## 1. Introduction

More than 80% of all plant and animal species on Earth can be found in the oceans, along with an astounding variety of microbes. This exceptionally high level of biodiversity and chemodiversity, often substantially different from terrestrial ones, is explained by considering different factors; among these, the temperatures, which can vary greatly (from 35.0 °C to 1.5 °C), are of pivotal importance [1,2].

The marine environment has recently yielded a large number of bioactive compounds (e.g., peptides, alkaloids, macrocyclic lactones), offering a novel source of medications to treat serious illnesses such as inflammation and cancer [3]. Some marine-derived drugs have already been released into the market, while preclinical or early clinical development is underway for many other medicinal compounds from marine sources.

Despite growing interest, several challenges remain. Exploring deep-sea ecosystems requires specialised equipment and technology, making the process both expensive and logistically demanding. Consequently, most research still focuses on marine organisms found in shallow waters, leaving vast areas of marine biodiversity largely unexplored. Furthermore, the overharvesting of marine species for scientific purposes can compromise the integrity of fragile ecosystems and harm sensitive species, thereby raising significant ethical and environmental concerns.

Many marine organisms are also complex to cultivate or sustain in laboratory settings, which limits the ability to reproduce their bioactive compounds.

Lastly, international regulations and biodiversity agreements (such as the Nagoya Protocol) can further restrict access to marine genetic resources. Given the importance of these species and the difficulty in isolating them from the sea, approved products are commonly produced through synthetic processes.

**Figure 1:**
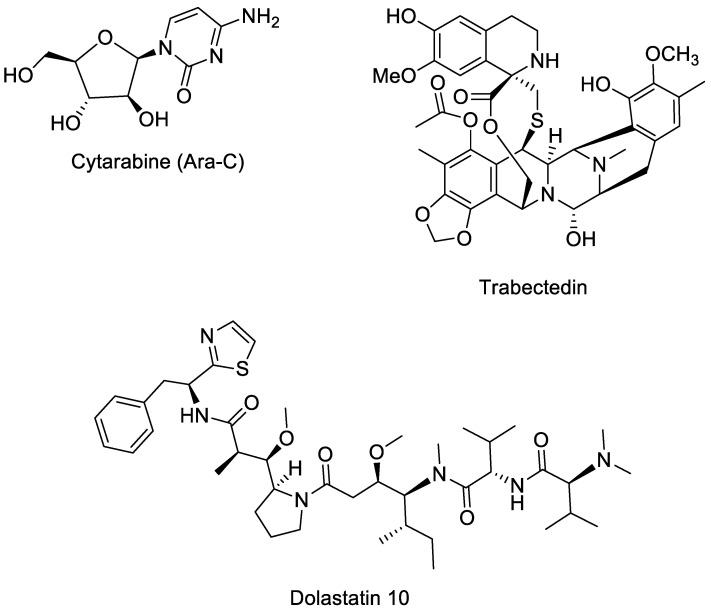
Structure of Cytarabine (Ara-C), Trabectedin, and Dolastatin 10.

The first FDA-approved drug derived from a marine source was Cytarabine (Ara-C, Figure 1), approved in 1969 [4]. It was initially isolated from the marine sponge *Cryptotethya crypta* and is an analogue of cytidine that combines a cytosine base with an arabinose sugar [5]. Its discovery marked the beginning of drug development from marine environments. Cytarabine is primarily used to treat various forms of leukaemia, including acute myelocytic leukaemia, lymphocytic leukaemia, meningeal leukaemia, and the blast crisis phase of chronic myelogenous leukaemia [6,7,8,9]. Even today, it remains a key chemotherapeutic agent in clinical practice. Its success laid the foundation for the continued exploration of the oceans as a valuable source of new pharmaceuticals.

Since then, numerous compounds derived from marine sources have been approved. Trabectedin (Figure 1), an alkaloid originally derived from the sea squirt *Ecteinascidia turbinate* [10], was the first marine-derived anticancer drug approved in the EU (2007) and later by the FDA (2015) for soft tissue sarcoma and ovarian cancer [11].

Dolastatin 10 (Figure 1) is an anticancer peptide derived from the sea hare *Dolabella auricularia* [12] and is currently in phase I clinical trials. It strongly inhibits the growth of human prostate cancer DU-145 cells in culture at very low concentrations (IC_50_ = 0.5 nM), causing cell cycle arrest in the G2/M phase and depolymerization of α-tubulin [13]. In animal studies, treatment with 5 μg every four days prevented tumour invasion in mice. Dolastatin 10 is therefore a promising marine-derived compound for prostate cancer therapy, acting through tubulin depolymerization rather than apoptosis induction [13].

Moreover, numerous Antibody Drug Conjugates inspired by marine peptides were approved by EMA and FDA for different treatments (e.g., polatuzumab vedotin (Polivy), enfortumab vedotin (Padcev), etc.) [14].

**Figure 2:**
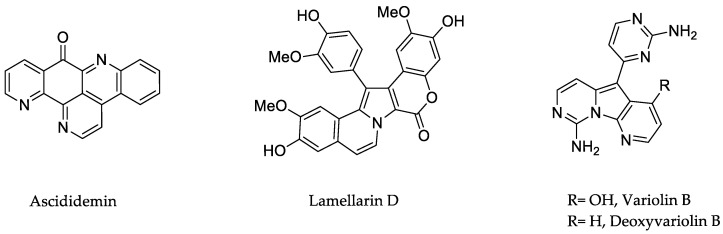
Structure of ascididemin, lamellarin D, variolin B and deoxyvariolin B.

**Figure 3:**
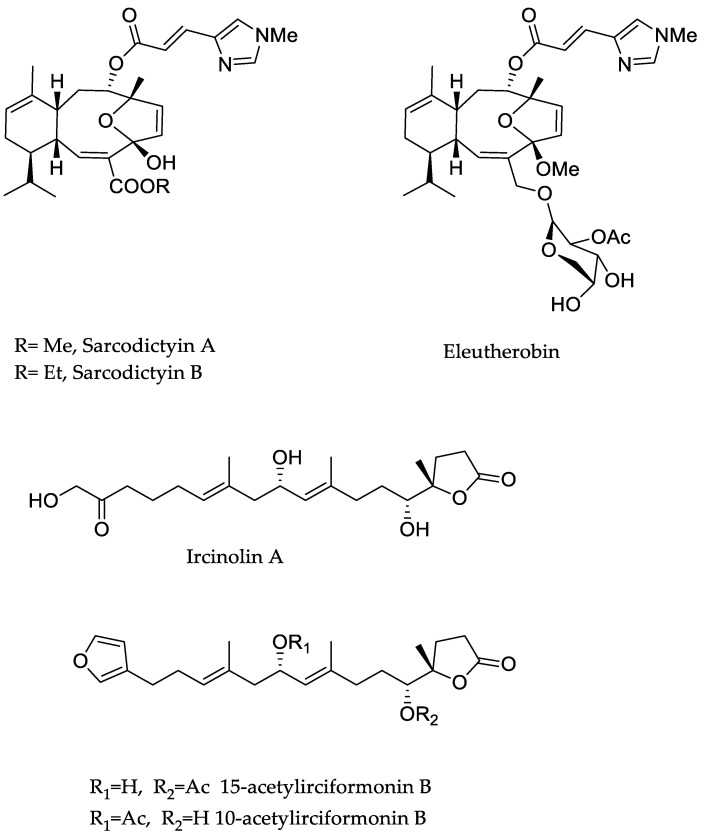
Structure of sarcodictyins, eleutherobin, 15-acetylirciformonin B and 10-acetylirciformonin B.

**Figure 4:**
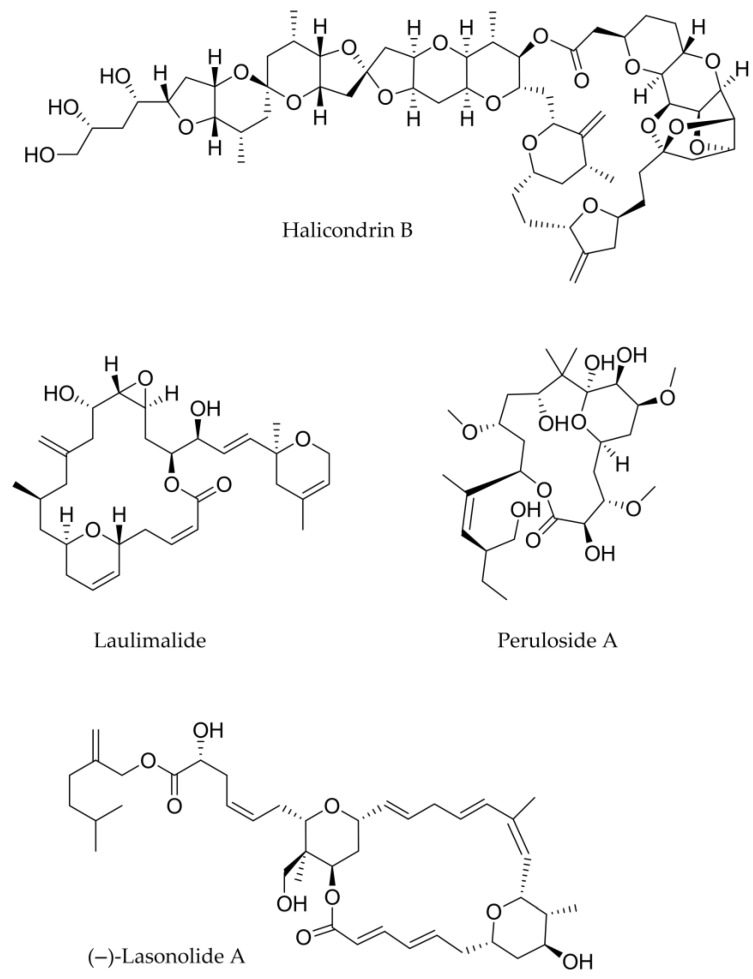
Structure of halichondrin B, laulimalide, peloruside A and lasonolide.

**Figure 5:**
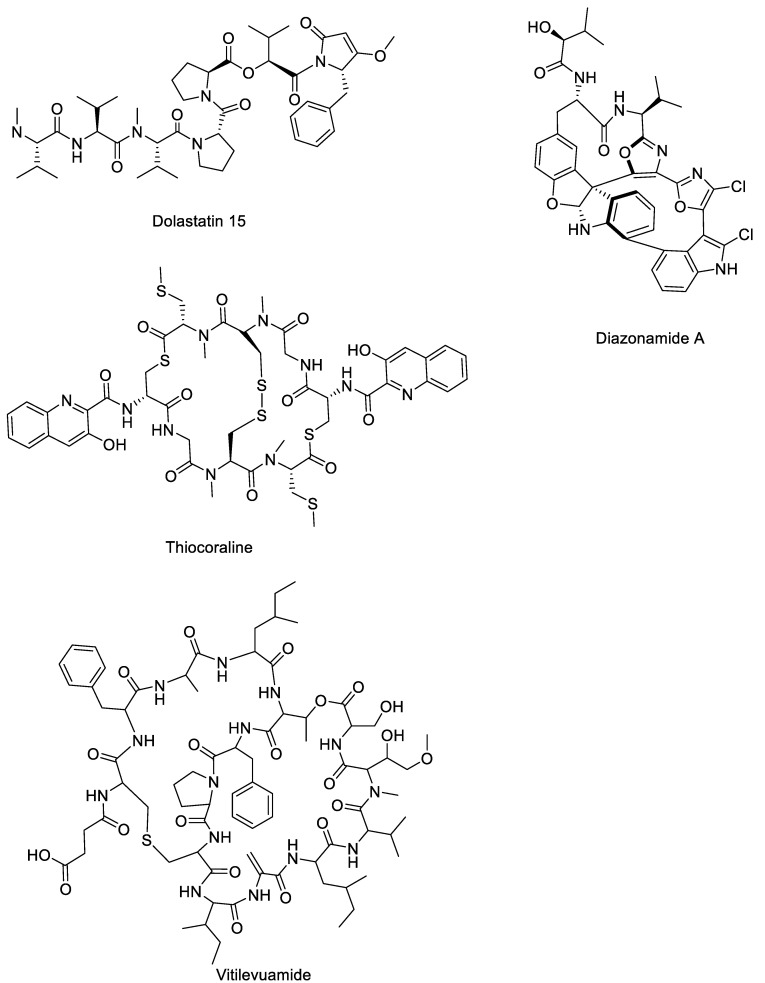
Structure of dolastatins [91], diazonamide [94], thiocoraline [99], and vitilevuamide [104].

**Figure 6:**
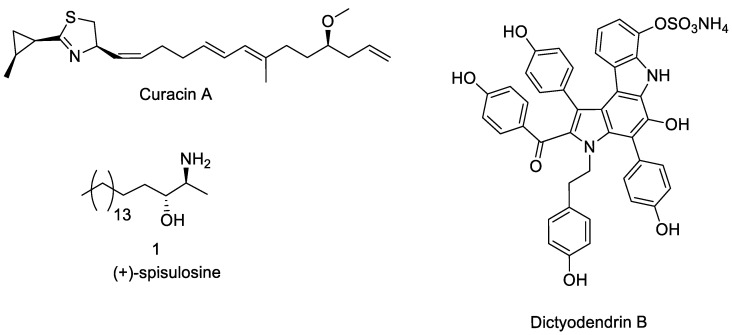
Structure of curacin A, spisulosine, and dictyodendrins.

While Cytarabine, Trabectedin, and Dolastatin 10 are either approved drugs or currently in clinical trials, this review focuses specifically on marine-derived compounds that are in preclinical studies as anticancer agents. Emphasis is placed on their unique chemical structures and on the total synthesis approaches that have been optimised over the last decade, highlighting their promising therapeutic potential and the opportunities they offer for future drug development. These compounds of marine origin are of great importance for drug development; however, their limited availability from natural sources restricts their use. Therefore, chemical synthesis is necessary to ensure an adequate supply. These marine-derived compounds are classified in different classes: aromatic and heterocyclic alkaloids (Table 1 and Figure 2); terpenes and derivatives (Table 2 and Figure 3); macrolydes (Table 3 and Figure 4); linear, cyclic and depsipeptides (Table 4 and Figure 5) and other classes (e.g., polyketide, thiazole lipid, alkylamino alcohol, pyrrolocarbazole derivatives) (Table 5 and Figure 6).

## 2. Marine Natural Products in Preclinical Cancer Research

### 2.1. Aromatic and Heterocyclic Alkaloids

Ascididemin (Figure 2) is the first marine-derived pyridoacridine alkaloid, isolated in 1988 [15]. Originally obtained from the ascidian *Didemnum* sp., it has strong anticancer properties. Ascididemin inhibits topoisomerase II [16], an enzyme essential for DNA replication and repair, by intercalating into DNA, leading to DNA damage, cell death, and suppression of cancer cell growth. Ascididemin has been shown to exhibit cytotoxic effects against a variety of cancer cell types (HL-60, MCF7, HCT-116, and A549) with IC_50_ values ranging from nanomolar to micromolar [16,17]. Additionally, Vollmar’s group demonstrated that ascididemin causes leukaemia Jurkat T cells to undergo apoptosis through a signalling cascade that necessitates the activation of the initiator caspase-2 upstream of mitochondria. Ultimately, in the same work, its cytotoxicity was demonstrated even against cancer cells that were resistant to many drugs [18].

In 1985, Faulkner and coworkers reported the first isolation of the lamellarin class marine alkaloids, lamellarins A, B, C, and D, from the prosobranch mollusk, *Lamellaria* sp. Since then, more than 50 lamellarins have been isolated from several marine organisms [19]. Among these aromatic and heterocyclic alkaloids, lamellarin D (Figure 2) is a potent anticancer compound. By inhibiting topoisomerase I, it exhibited mitochondrial pro-apoptotic activity, with in vitro cytotoxicity against various tumour cell lines (IC_50_ < 1 µM), including drug-resistant models [20]. In vivo, it demonstrated anticancer activity by slowing the growth of tumours in murine xenograft models. Among the series of natural compounds, lamellarin D is one of the most powerful [21], and it seems to be more well-tolerated than similar substances like ascididemin; nevertheless, comprehensive information on pharmacokinetics and systemic toxicity is still scarce [22].

In Antarctica, the sponge *Kirkpatrickia variolosa* is the source of the unique marine natural substance variolin B (Figure 2) [2]. Given its demonstrated pro-apoptotic properties, variolin B was a novel and highly effective natural cytotoxic agent [23]. Nevertheless, the poor stability of variolin B in solution restricts its application. This drawback of the original molecule has been addressed by the creation of a deoxyvariolin B (Figure 2) analogue that is far more stable and soluble. The biological activity of this deoxy analogue was comparable to that of variolin B, with a modest increase in potency that is probably due to its increased solubility and stability [24].

Both variolin B and deoxy variolin [25], show potent cytotoxic effects on a variety of tumour cell lines (IC_50_ in the 50–100 nM range). Due to their cytotoxic activities, Simone et al. [26] determined the molecular target of these substances. Their studies revealed that cyclin-dependent kinases (CDKs) are the main targets of these compounds, and consistent with their cell cycle-blocking properties, they preferentially inhibit the phosphorylation of histone H1 mediated by CDK1 and CDK2, but also inhibit CDK4 and CDK7.

Then, Meijer and colleagues [27] additional investigations supported this CDK inhibition profile and demonstrated that variolin B inhibits CDK9 even more potently than CDK1 or CDK2 (IC_50_ = 26 nM). Thus, its peculiar heterocyclic structure became a new scaffold for creating novel CDK inhibitors, and deoxy-variolin B was selected for preclinical development by PharmaMar due to its improved physicochemical properties over variolin B, including better solubility and stability [24,28]. Structural analogues (≥16 compounds) with modifications at positions C5 and C7 were synthesised; some of these analogues displayed antiproliferative activity comparable to or greater than variolin B across several cancer cell lines (colon, breast, melanoma, ovary, lung, pancreas, etc.) [28].

There are several methods to synthesise variolin B and deoxy variolin B. The first process is an 8-step procedure that begins with 4-chloro-2-methylthiopyrimidine and yields a 17% overall yield [24]. In 2008, Baeza et al. [29] introduced a new method based on selective Pd-catalysed C–N, C–C, and C–O bond formations on a trihalo-substituted pyridopyrrolopyrimidine intermediate to build the variolin B framework. In 2010, the same group published a new, more efficient route for synthesising variolins and potential analogues through a selective palladium-mediated functionalization of the pyrido [3′,2′:4,5]pyrrolo[1,2-*c*]pyrimidine core to rapidly install key substituents [30]. However, we will not go into greater detail about these works in this review since this topic was not further investigated in published papers in the last 10 years.

**Table 1:** Marine-derived aromatic and heterocyclic alkaloids with anticancer activities: chemical classification, source and involvement in cancer.

Compound (Isolation)	Class	Source	Cancer Type
Ascididemin (1988)	Aromatic alkaloid	*Didemnum* sp. (sponge)	Leukaemia
Lamellarin D (1985)	Pyrrole alkaloid	*Lamellaria* sp. (mollusc/corals)	Leukaemia, colon cancer
Variolins (1994)	Heterocyclic alkaloid	*Kirkpatrickia variolosa* (sponge)	Leukaemia, brain tumours

#### Lamellarin D Total Syntheses

**Scheme 1:**
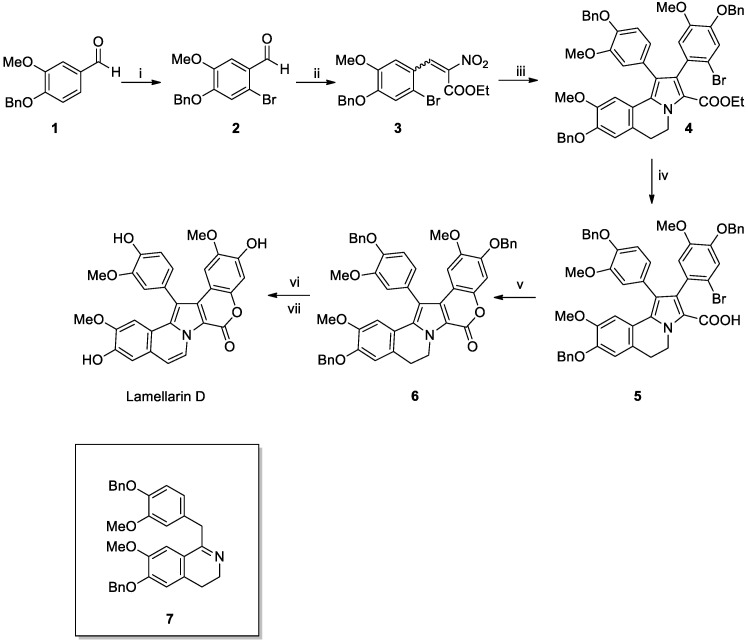
Synthesis of lamellarin D reported by Chittchang’s group in 2015 [37]. Reagents and conditions: (i) PyHBr_3_, MeOH, r.t., 3 h; (ii) ethyl 2-nitroacetate, Et_2_NH·HCl, toluene, reflux, Ar (g), 3 days; (iii) **7**, NaHCO_3_, MeCN, reflux, O/N; (iv) NaNH_2_, anh. 1,4-dioxane, 100 °C, 18 h; (v) CuTC, DMF, MW (200 W, 150 °C), 45 min; (vi) DDQ, CH_2_Cl_2_, r.t., 4 h; (vii) TFA, thioanisole, r.t., 6 h.

The first total synthesis of lamellarin D was accomplished by Iwao and co-workers in 1997 [31]. Over the years, numerous total syntheses of lamellarin D have been performed [32,33,34,35,36]. In 2015, Chittchang [37] and coworkers published a common general approach to lamellarin D and azalamellarin D (Scheme 1). Starting from 4-(benzyloxy)-3-methoxybenzaldehyde (**1**), the bromination with pyridinium tribromide gave compound **2**, which reacted with ethyl 2-nitroacetate, in the presence of Et_2_NH·HCl, in toluene at reflux, to give compound **3**. This underwent a crucial Michael reaction with benzyl dihydroisoquinoline **7**, producing the 2-carboethoxypyrrole intermediate **4**. The pyrrole carboxylic acid (compound **5**) was unexpectedly obtained by the attempted amination of the pyrrole ester using NaNH_2_, in dioxane at 100 °C. The resulting acid was then cyclized to form a lactone via microwave-assisted intramolecular C–O bond formation in the presence of copper(I) thiophene-2-carboxylate (CuTC). Subsequently, a global DDQ-mediated oxidation was performed, followed by a TFA-mediated acidolysis in thioanisole, which efficiently removed all -OBn-type protecting groups in a single step, affording the lamellarin D in excellent yield.

**Scheme 2:**
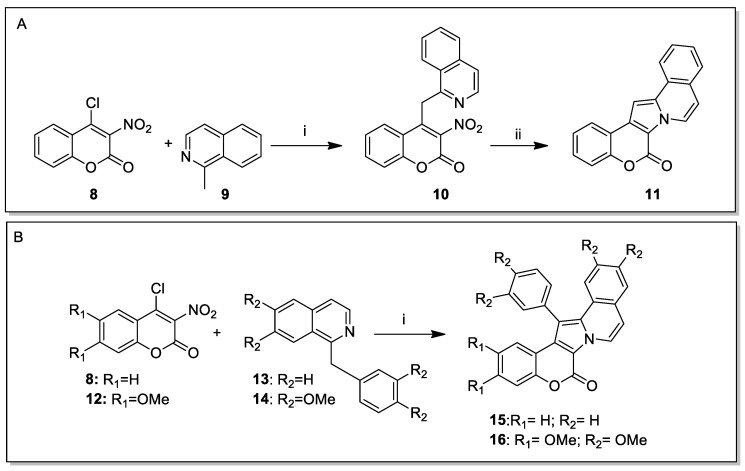
(**A**) Synthesis of compound **11** [38]. Reagents and conditions: (i) K_2_CO_3_, DCE, reflux, 12 h; (ii) visible light, acetonitrile, 12 h; (**B**) Synthesis of lamellarian core [38]. Reagents and conditions: (i) cat Yb(OTf)_3_, xylene, 130 °C, 2 days.

Yang and coworkers reported two different methodologies for synthesizing the lamellarin core [38]. In 2016, they reacted 4-chloro-3-nitrocoumarin (**8**) and 1-methylisoquinoline (**9**) in basic conditions to give 4-(isoquinolin-1-ylmethyl)-3-nitro-2*H*-chromen-2-one (**10**), which was converted to **11** by visible-light-mediated cyclisation reaction. Unfortunately, this procedure failed when applied to 1-benzylisoquinoline; therefore, they opted for a direct coupling of **8** and **13**, catalysed by Yb(OTf)_3_ Lewis acid in xylene under reflux, to give the lamellarin core **15**. This optimised methodology was then utilised in the preparation of lamellarin D trimethyl ether (**16**), by coupling 4-chloro-6,7-dimethoxy-3-nitrocoumarin (**12**) with commercially available papaverine (**14**) to give lamellarin D trimethyl ether (**16**) in 8% overall yield in three steps (Scheme 2). Subsequent exhaustive demethylation of **17** using boron tribromide afforded the natural product lamellarin H.

**Scheme 3:**
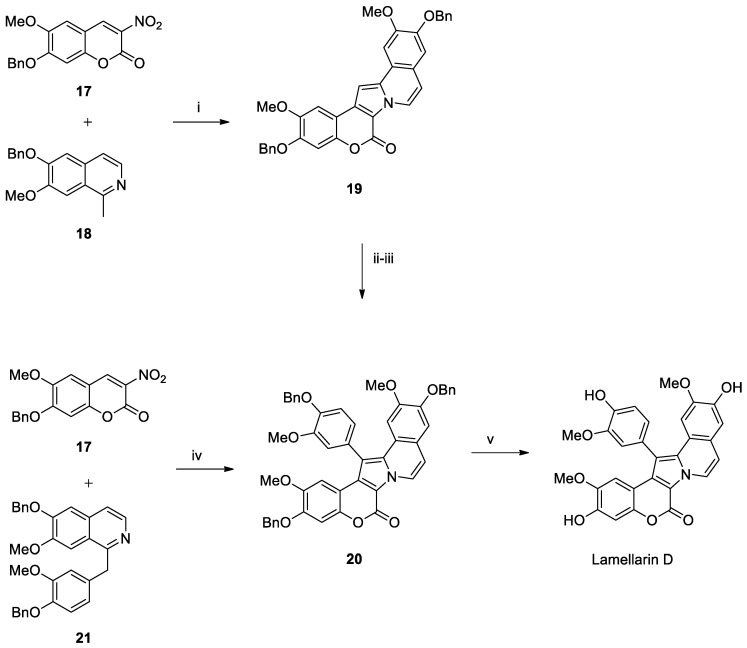
Synthesis of lamellarin D reported by Yang’s group in 2017 [39]. Reagents and conditions: (i) NaHCO_3_, xylene, sealed tube, 120 °C, 18 h; (ii) NBS, THF, 0 °C-r.t., overnight; (iii) (4-(benzyloxy)-3-methoxyphenyl)boronic acid, CSF, Ag_2_O, Pd(PPh_3_)_4_, DME, reflux, 24 h; (iv) NaHCO_3_, xylene sealed tube, 130 °C, 24 h; (v) H_2_, Pd(OH)_2_/C, AcOEt, r.t., 8 h.

In a subsequent work, the same group described the synthesis of lamellarin D (Scheme 3) along with that of lamellarin H [39]. The Grob coupling reaction between 3-nitrocoumarin **17** and 1-methylisoquinoline **18** was employed for the synthesis of the key intermediate **19** by using NaHCO_3_ and xylene in a sealed tube at 120 °C for 18 h. The building block was subsequently brominated and then coupled with [4-(benzyloxy)-3-methoxyphenyl]boronic acid to give the intermediate **20**. Finally, the debenzylation mediated by Pd(OH)_2_ and AcOEt afforded the lamellarin D product. Moreover, in the same work, the authors demonstrated how to obtain intermediate **20** through a direct coupling between 3-nitrocoumarin **17** and benzylisoquinoline **21** under similar coupling conditions, by using NaHCO_3_ and xylene in a sealed tube, this time at 130 °C for 24 h. This work represents the shortest route reported to date to obtain the lamellarin D.

**Scheme 4:**
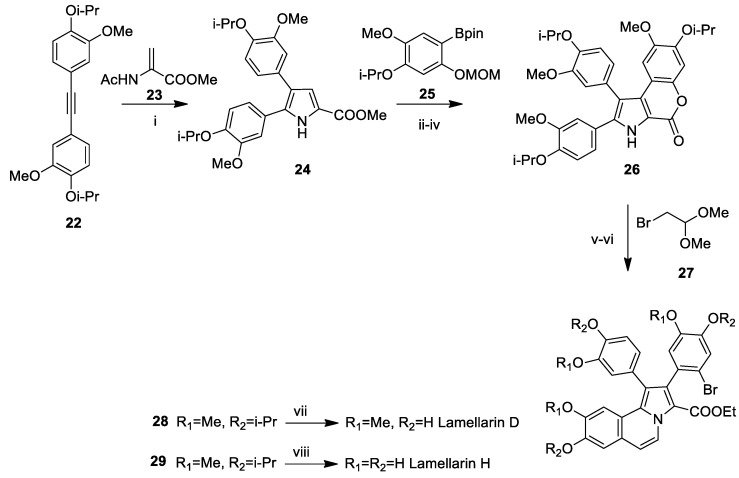
Synthesis of lamellarin D reported by Chandrasekhar’s group in 2017 [40]. Reagents and conditions: (i) [RuCl_2_(p-cymene)]_2_, Cu(OAc)_2_⋅H_2_O PEG-400, 110 °C, 24 h; (ii) NBS, DMF, 0 °C to r.t., 12 h; (iii) **25**, Pd_2_(dba)_3_, dppf, DME/H_2_O, 110 °C, 36 h; (iv) pTsOH, MeOH, 16 h, 110 °C; (v) **27**, Cs_2_CO_3_, DMF, 110 °C, 24 h; (vi) TfOH, CH_2_Cl_2_, 0 to 24 °C, 1.5 h; (vii) BCl_3_, CH_2_Cl_2_, –78 to 24 °C, 3.5 h; (viii) BBr_3_, CH_2_Cl_2_, –78 to 24 °C, 17 h.

In 2017, Chandrasekhar’s group synthesised lamellarin D (Scheme 4) [40]. The procedure began with compound **22**. By using the [RuCl_2_(*p*-cymene)]_2_ and AgSbF_6_, in the presence of Cu(OAc)_2_·H_2_O as the oxidant, in PEG-400, the diarylpyrrole-5-carboxylate **24** was obtained. Afterwards, the obtained pyrrole **24**, once brominated, was coupled by a Suzuki–Miyaura reaction, giving the tetrasubstituted pyrrole **26**, which was subjected to a Pomeranz–Fritsch reaction to create the lactone ring, affording the desired lamellarins H and D, with an overall yield of 29% and 37%, respectively.

Almost simultaneously, Ackermann’s group published a synthesis of lamellarin D and H [41] using the same procedure described by Chandrasekhar’s group (Scheme 4). In this case, Ackermann’s group took advantage of the recent advancements in the application of Ru(II) catalysts for C–H/N–H activation. The central pyrrol ring of the alkaloids was obtained by a Ru-catalysed (3 + 2) oxidative annulation between enamide **23** with alkyne **22**. The optimisation of reaction conditions resulted in a gram-scale synthesis of intermediate **24** (Scheme 4).

**Scheme 5:**
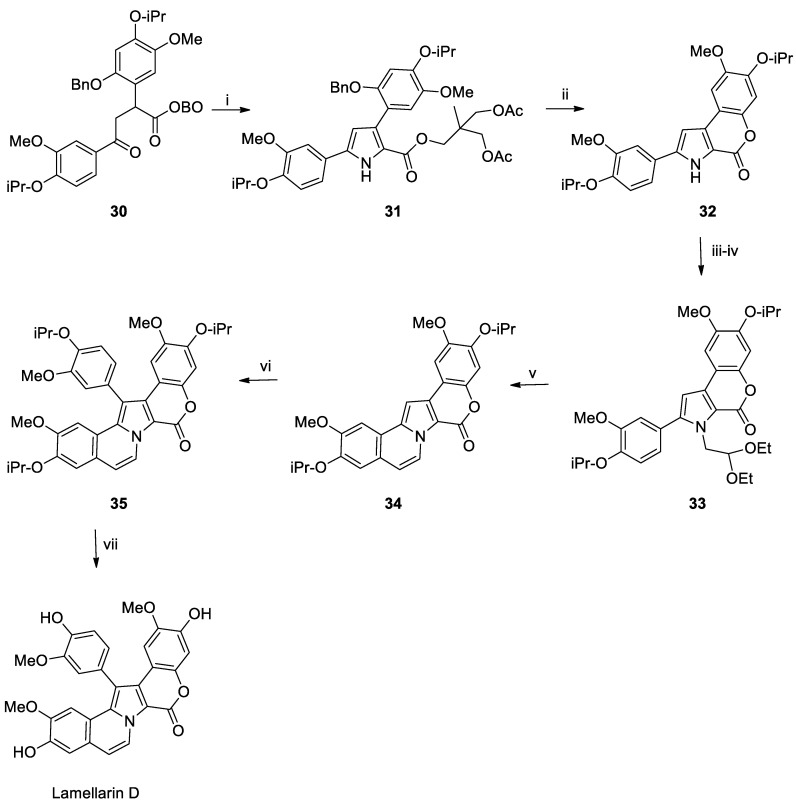
Synthesis of lamellarin D reported by Shirley et al. in 2019 [42]. Reagents and conditions: (i) NH_4_OAc, AcOH, 110 °C, 30 min; (ii) H_2_, Pd/C, EtOH, rt, 6.5 h; (iii) K_2_CO_3_, EtOH, 90 °C, 2 h; (iv) BrCH_2_CH(OEt)_2_, Cs_2_CO_3_, DMF, 110 °C, 24 h; (v) TfOH, DCM, −10 °C to −5 °C, 24 h; (vi) Pd(PPh_3_)_2_Cl_2_ (5 mol%), KOAc, 4-bromo-1-isopropoxy-2-methoxybenzene, DMA, 150 °C, 22 h; (vii) BCl_3_, DCM, −78 °C to rt, 3.5 h. OBO= oxabicyclo [2.2.2]octyl orthoester.

In 2019 Shirley et al. published [42] a chemical synthesis procedure (Scheme 5) starting from compound **30** that was cyclized to obtain the pyrrole ring by using an excess of NH_4_OAc in acetic acid at 110 °C for 30 min. The debenzylation of intermediate **31** involves the subsequent processes, which include lactonisation with K_2_CO_3_ in ethanol to obtain compound **32**. Compound **33** was then produced by alkylating the pyrrole with bromoacetaldehyde acetate, which, by eliminating the acetal intermediate in situ using catalytic TfOH, gave compound **34**. The last synthesis steps involved CH-arylation of molecule **34** with previously synthesised 4-bromo-1-isopropoxy-2-methoxybenzene, followed by the selective deprotection of the isopropoxy groups using BCl_3_, yielding lamellarin D.

This approach was very innovative because bicyclic ring pyrrolo-benzofurans are obtained in a single system from a 1,4-dicarbonyl intermediate. Moreover, it was the first time that the C-H arylation on a late pyrrole was applied in the total synthesis of a lamellarin. The direct arylation reaction catalysed by Pd(II) was performed without the need for pre-activation of the pyrrole (e.g., bromuration followed by coupling), overcoming the limitations of the classical strategies based on halogenation and Suzuki/Stille coupling.

### 2.2. Terpenes and Derivatives

Among terpenes and their derivatives, sarcodictyins are a family of cembranoid diterpenes isolated from soft corals of the genus *Sarcodictyon* [43]. Sarcodictyin B has also demonstrated some antimitotic activity [44]; however, sarcodictyin A (Figure 3 and Table 2) remains the reference compound for preclinical studies. It demonstrated antitumor activity [45] in preclinical models. Its mechanism of action is similar to that of paclitaxel (Taxol), acting by stabilising microtubules and thereby preventing cell division (mitosis). Sarcodictyin A was studied in vitro and in animal models for the treatment of various cancers, including breast cancer, ovarian cancer, and solid drug-resistant tumours [46,47].

Eleutherobin (Figure 3 and Table 2) is another naturally occurring terpene that stabilises microtubules. Initially identified from the rare soft coral *Eleutherobia* sp. in 1997 in Western Australia [48], it contains a central nine-membered unsaturated heterocyclic ring, showing an antitumor potency ten times greater than those of Taxol and Docetaxel [44,48].

Eleutherobin demonstrated potent cytotoxic activity against multiple tumour cell lines [49], and it showed nearly 100-fold greater potency against lung, ovarian, kidney, and breast cancer cells, as evaluated in a screening made by The National Cancer Institute (NCI) against 60 cancer cell lines. Finally, the NCI analysis revealed that its tumour selectivity pattern substantially matches that of paclitaxel [48]. Due to its antitumor properties, significant efforts were made toward the total synthesis of this marine natural compound.

In 2011, Su et al. [50] isolated ircinolin A, 15-acetylirciformonin B, and 10-acetylirciformonin B from the marine sponge *Ircinia* sp. Ircinolin A is the first metabolite generated from C_21_ terpenoid identified in *Ircinia*. In that same paper [50], two novel C_22_ furanoterpenoid metabolites were identified: 15-acetylirciformonin B and 10-acetylirciformonin B. The cytotoxicity of each of these substances was evaluated against several cancer cell lines, including the leukaemia line K562, the colon cancer cell line DLD-1, and the liver cancer cell lines HepG2 and Hep3B. In these screening tests, 15-acetylirciformonin B was the most effective of all. IC_50_ values were 0.03 µM (DLD-1), 0.5 µM (HepG2), 1.1 µM (Hep3B), and approximately 5.4 µM (K562). No complete synthesis of ircinolin A, 15-acetylirciformonin B, or 10-acetylirciformonin B has been documented as of the most recent literature.

**Table 2:** Marine-derived terpenes and derivatives with anticancer activities: chemical classification, source, and involvement in cancer.

Compound (Isolation)	Class	Source	Cancer Type
Sarcodictyins (1987s)	Diterpene	*Sarcodictyon roseum* (sponge)	Ovarian, breast cancer, solid tumours
Eleutherobin 1997	Diterpene glycoside	*Eleutherobia* sp./*Erythropodium* (sponge)	Leukaemia, breast cancer
Ircinolin A 2011	*N*orsesterterpenoid	*Sponge Ircinia* sp. (sponge)	Leukaemia, colon cancer, hepatocellular carcinoma
15-acetylirciformonin B 2011	Furanosesterterpenoids	*Sponge Ircinia* sp. (sponge)	Leukaemia, colon cancer, hepatocellular carcinoma
10-Acetylirciformonin B 2011	Furanosesterterpenoids	*Sponge Ircinia* sp. (sponge)	Leukaemia, colon cancer, hepatocellular carcinoma

#### Recent Synthesis of Sarcodictyins and Eleutherobin

K.C. Nicolaou and colleagues were the first to complete a total synthesis of sarcodictyins A and B [51]. They developed two distinct approaches to access both compounds, starting from (+)-carvone, completing the synthesis in 29 linear steps. Since then, no new total syntheses of sarcodictyins have been published until 2025.

**Scheme 6:**
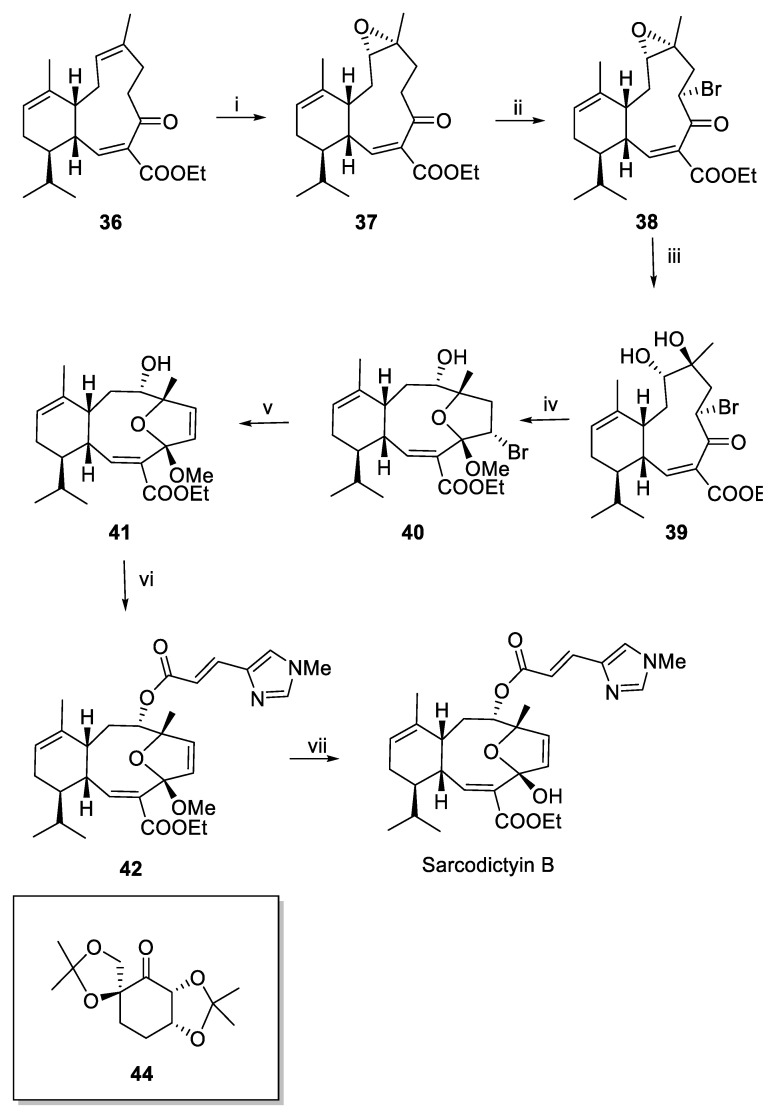
Synthesis of sarcodictyins reported by Britton’s group in 2025 [52]. Reagents and conditions: (i) Compound **44**, oxone, K_2_CO_3_, Bu_4_N(HSO_4_), Na_2_EDTA, Na_2_B_4_O_7_·H_2_O, MeCN; DMM, H_2_O, r.t.; (ii) LiHMDS, NEt_3_, TMSCl, NBS, THF, −78° to −10 °C; (iii) TsOH H_2_O, MeCN/H_2_O, 70 °C; (iv) PPTS, MeOH, 50 °C; (v) Co(dmgH)_2_PyCl, 4CzIPN, DIPEA, MeCN, blu LEDs, r.t.; (vi) (*E*)-3-(1-methyl-1*H*-imidazol-4-yl)acrylic acid, DCC, DMAP, CDCl*_3_*, 50 °C; (vii) CSA, CH_2_Cl_2_, H_2_O, r.t.

**Scheme 7:**
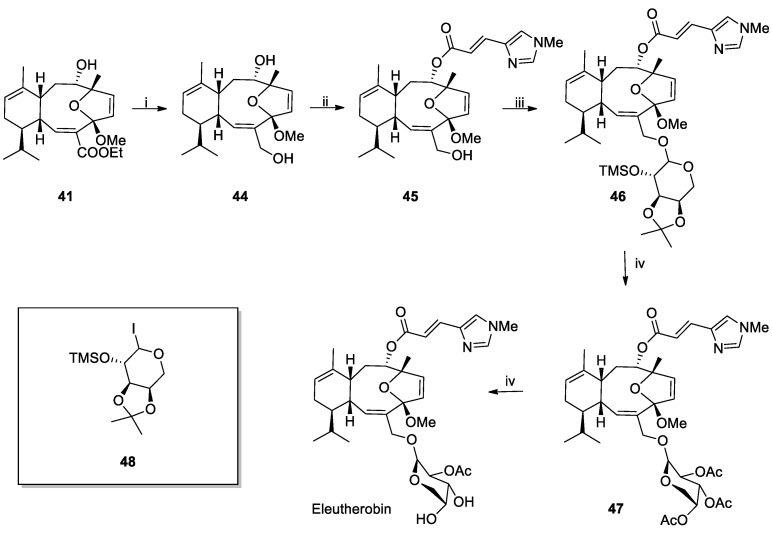
Synthesis of eleutherobin reported by Britton’s group in 2025 [52]. Reagents and conditions: (i) DIBAL-H, CH_2_Cl_2_, −78 °C; (ii) DCC, DMAP, CHCl_3_, 3 °C, (*E*)-3-(1-methyl-1*H*-imidazol-4-yl)acrylic acid; (iii) **48**, Ac_2_O, pyridine, DMAP, (iv) PPTS, MeOH, reflux.

Very recently, the Britton group published a new synthesis of sarcodictyins and eleutherobin (Scheme 6 and Scheme 7) [52]. Their synthesis of sarcodictyins (Scheme 6) began from intermediate **36**, also known as eunicellin. After several trials, the C7–C8 alkene was *si-*face epoxidised with remarkable selectivity using the fructose-derived catalyst (compound **43**), yielding compound **37** as a single diastereomer. Subsequently, bromination at position 3 was achieved using *N*-bromosuccinimide (NBS), which reacted in situ with lithium bis(trimethylsilyl)amide (LiHMDS) in the presence of NEt_3_ and TMSCl. In the next step, epoxide ring opening yielded a diol (**39**), in which the hydroxy group at position 7 reacted with the carbonyl at position 4 to form a hemiacetal group, characteristic of compound **40**. This crucial step was achieved by Leonori photochemical halogen atom transfer reaction by using the photocatalyst 4CzIPN, diisopropylethylamine, and a cobalt(III) cocatalyst. The desired alkene **41** was then obtained in good yield using the Leonori photochemical catalyst [53]. Finally, the reaction of compound **41** with (*E*)-3-(1-methyl-1*H*-imidazol-4-yl)acrylic acid produced sarcodictyin B methyl acetal (**42**), which was subsequently converted to sarcodictyin B through hydrolysis of the methyl acetal.

The groups of Nicolaou and Danishefsky reported the first two total syntheses of eleutherobin [54,55,56], after which other methods for synthesising have been reported over the years [57,58]. The last work on the synthesis of eleutherobin dates back to Britton’s group, in the same work in which the synthesis of sarcodictyins is reported [52]. The synthesis of eleutherobin (Scheme 7) and its analogues was investigated, successfully reducing the number of linear steps to 18 compared to previous syntheses requiring 26–28 steps. Starting from the same intermediate **41,** the ethyl ester at position 15 was first reduced using DIBAL-H in CH_2_Cl_2_ at −78 °C. This was followed by selective esterification at C8 with (*E*)-3-(1-methyl-1*H*-imidazol-4-yl)acrylic acid to afford intermediate **45**.

Then, a glycosyl iodide (**48**) solution (previously obtained from the TMS-protected sugar and trimethylsilyl iodide) reacted with compound **45** using 2,6-di-tert-butylpyridine as a base in CH_2_Cl_2_ at room temperature. The resulting glycosylated product (**46**) was peracetylated using acetic anhydride and DMAP (**47**). Finally, deprotection with PPTS in refluxing methanol liberated the 3′- and 5′-hydroxyl groups, affording the fully elaborated eleutherobin.

### 2.3. Macrolides

Isolated from *Halichondria* sponges, halichondrin B (Figure 4 and Table 3) is a complex polyether macrolide with marine origins [59]. Halichondrin B primarily exerts its activity by inhibiting the formation of microtubules, which are necessary for mitosis and other biological functions [60]. Despite its potency, halichondrin B has a very low natural abundance, making it challenging to extract large amounts from natural sources for in-depth study and therapeutic use. Because of this paucity, chemists worked to fully synthesise halichondrin B and its analogues. Notably, its synthetic analogue eribulin (E7389) was approved by the FDA as an anticancer medication for liposarcoma and breast cancer [61].

Laulimalide (Figure 4 and Table 3) was first discovered from the marine sponge Cacospongia mycofijiensis [62] and is a strong macrolide generated from marine sources with significant anticancer properties. The primary way in which laulimalide acts as an anticancer drug is by stabilising microtubules in a manner similar to paclitaxel [63,64], but at a different binding location on β-tubulin [65]. In light of this, it is effective against cancer cells that are resistant to paclitaxel because it does not compete with it for the binding site. Moreover, laulimalide shows nanomolar IC_50_ values against a broad panel of human cancer cell lines, including breast, colon, lung, and ovarian cancers [63].

(−)-Lasonolide A also belongs to this class of compounds (Figure 4 and Table 3). It was first isolated from the marine sponge *Forcepia* sp. and found in waters close to the Bahamas [66]. (−)-Lasonolide A exhibits nanomolar potency to a number of human cancer cell lines [67]. Zhang et al. [68] revealed the exceptional potency of lasonolide A, inducing a reversible chromosome condensation, acting independently of the mitotic CDK1 pathway and involving histone modifications and topoisomerase activation. In addition, the authors described the (−)-lasonolide A as a promising research tool and a potential anticancer lead, though its exact target and therapeutic window remain to be defined.

Additionally, peloruside A (Figure 4 and Table 3) was discovered in 2000 by Northcote and colleagues [69] from the marine sponge *Mycale hentscheli* and collected in Pelorus Sound, New Zealand. De Brabander’s [70] first complete synthesis revealed its absolute configuration; since then, several complete syntheses were reported [71,72,73] but none in the past decade. Regarding the biological effect, peloruside A is a powerful microtubule stabiliser; it binds to a unique site on the tubulin α,β-heterodimer and, for this reason, it may act synergistically with paclitaxel (needed to be optimised), docetaxel (Taxotere), epothilone A, and discodermolide B [74]. Studies in cultured cells showed enhanced antiproliferative and microtubule-stabilising effects when peloruside A was combined with these agents [74].

Moreover, additional studies revealed that peloruside A is very effective at stopping the growth of lung and P-glycoprotein-overexpressing breast tumours in vivo and, more importantly, was better-tolerated than doxorubicin or paclitaxel.

**Table 3:** Marine-derived macrolide compounds with anticancer activities: chemical classification, source, and involvement in cancer.

Compound (Isolation)	Class	Source	Cancer Type
Halichondrin B (1986)	Macrolide	*Halichondria okadai* (sponge)	Breast, lung
Laulimalide 1988	Macrolide	*Cacospongia mycofijiensis* (sponge)	Lung, ovarian
(−)-Lasonolide A 1994	Macrolide	*Forcepia* sp. *(sponge)*	Lung
			pancreas
Peloruside A 2003	Macrolide	*Mycale hentscheli* (sponge)	Non-small cell, lung, ovarian

#### Recent Synthesis of Halichondrin B and (−)-Lasonolide A

With more than 70 stages and sophisticated stereoselective techniques, the first complete synthesis of halichondrin B represents a significant turning point in organic chemistry, since this polyether macrolide features 32 stereocenters. In 1992, Yoon, S. K produced the first total synthesis of Halichondrin B [75]. Many other full syntheses of halichondrin B and other members of the family, such as norhalichondrin B and homohalichondrin B, have been published since then [76,77,78,79,80]. In 2021, Nicolau et al. [81] described a novel, versatile strategy for the synthesis of halichondrin B in 25 linear steps starting from commercially available reagents.

**Scheme 8:**
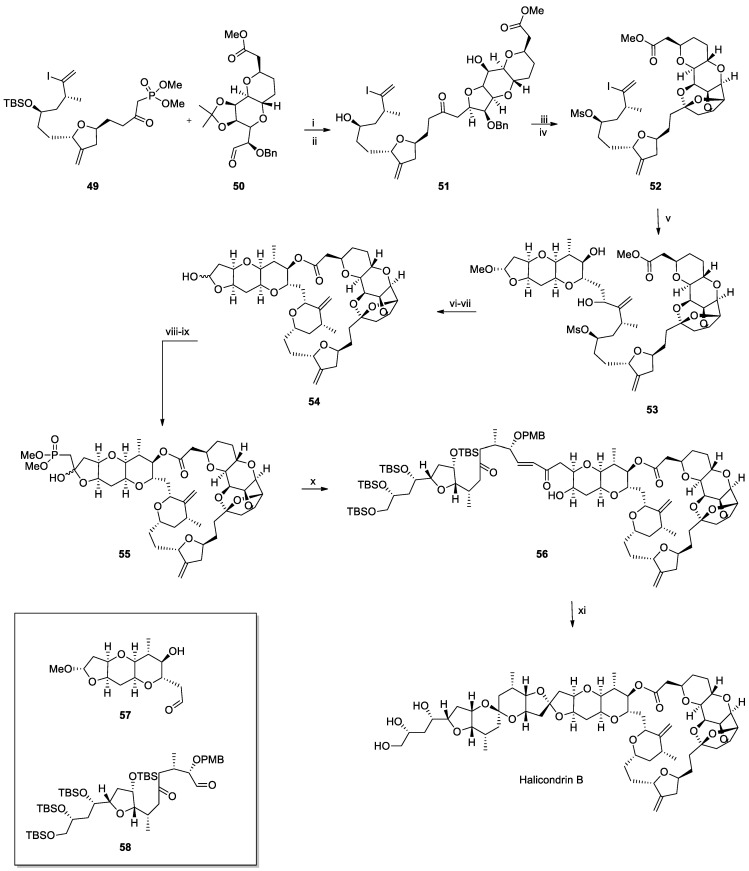
Synthesis of halicondrin B reported by Nicolau’s group in 2021 [81]. Reagent and conditions: (i) Et_3_N, LiCl; CH_3_CN, 0 °C to 23 °C; (ii) HF·Py (excess), CH_3_CN, 23 °C 3 h; (iii) hv, DDQ; (iv) MsCl, Et_3_N, DMAP, CH_2_Cl_2_, O °C, 15 min; (v) **57**, CrCl_2,_ NiCl_2_, Et_3_N, (*S*)-*N*-(2-(4-isopropyl-4,5-dihydrooxazol-2-yl)-6-methylphenyl)methanesulfonamide; (vi) KOH, 18-crown-6, then KOH, MeOH; (vii) MNBA, DMAP, i-Pr_2_NEt; (viii) PTSA·H_2_O; (ix) dimethyl (diazomethyl)phosphonate, SnCl_2_; (x) **58**, Et_3_N, LiBr, (xi) TBAF/AcOH, DDQ.

The authors began by synthesising all the key fragments. The synthesis of halichondrin B (Scheme 8) started with the coupling of fragments **49** and **50** (previously synthesised) mediated by NEt_3_ and LiCl; then, the use of a high concentration of HF permits the one-pot desilylation, the removal of the acetonide moiety, and the intramolecular cycloetherification to obtain the intermediate **51** as a mixture of its C12 diastereoisomer. Then, the addition of DDQ and light followed by MsCl in the presence of Et_3_N gave intermediate **52**. The latter are involved in the nucleophilic attack on the aldehyde group of compound **57**, achieved by using NiCl_2_, CrCl_2_, and Et_3_N, and by using (*S*)-N-(2-(4-isopropyl-4,5-dihydrooxazol-2-yl)-6-methylphenyl)methanesulfonamide as a catalyst. The intermediate **53** was subjected to hydrolysis and cycloesterification to obtain compound **54**. These steps are very critical and need to be optimised. The best conditions were achieved in KOH and 18-crown-6 in toluene/MeOH at 60 °C, and with the next addition of KOH (1M) in MeOH to afford the desired carboxylic acid. After the coupling between the carboxylic acid and the hydroxy group at position 3 on the pyran ring, the demethylation of the hydroxy group on the hydrofuran ring was achieved in the next step using PTSA. The final stages are represented by the formation of lactol phosphonate **55** (deriving from the reaction with dimethyl (diazomethyl)phosphonate and SnCl_2_) followed by the coupling with compound **58** in the presence of Et_3_N and LiBr. Finally, halichondrin B was obtained by cleaving the TBS and PMB protecting groups and cyclizing with DDQ to generate the cast ring of halichondrin B.

(−)-Lasonolide A shows a complex structure as well, and its complete synthesis has been a major achievement in organic chemistry. Asymmetric synthesis, macrolactonization, and convergent methods are employed in the various synthetic pathways that have been documented [82,83,84,85,86,87,88].

**Scheme 9:**
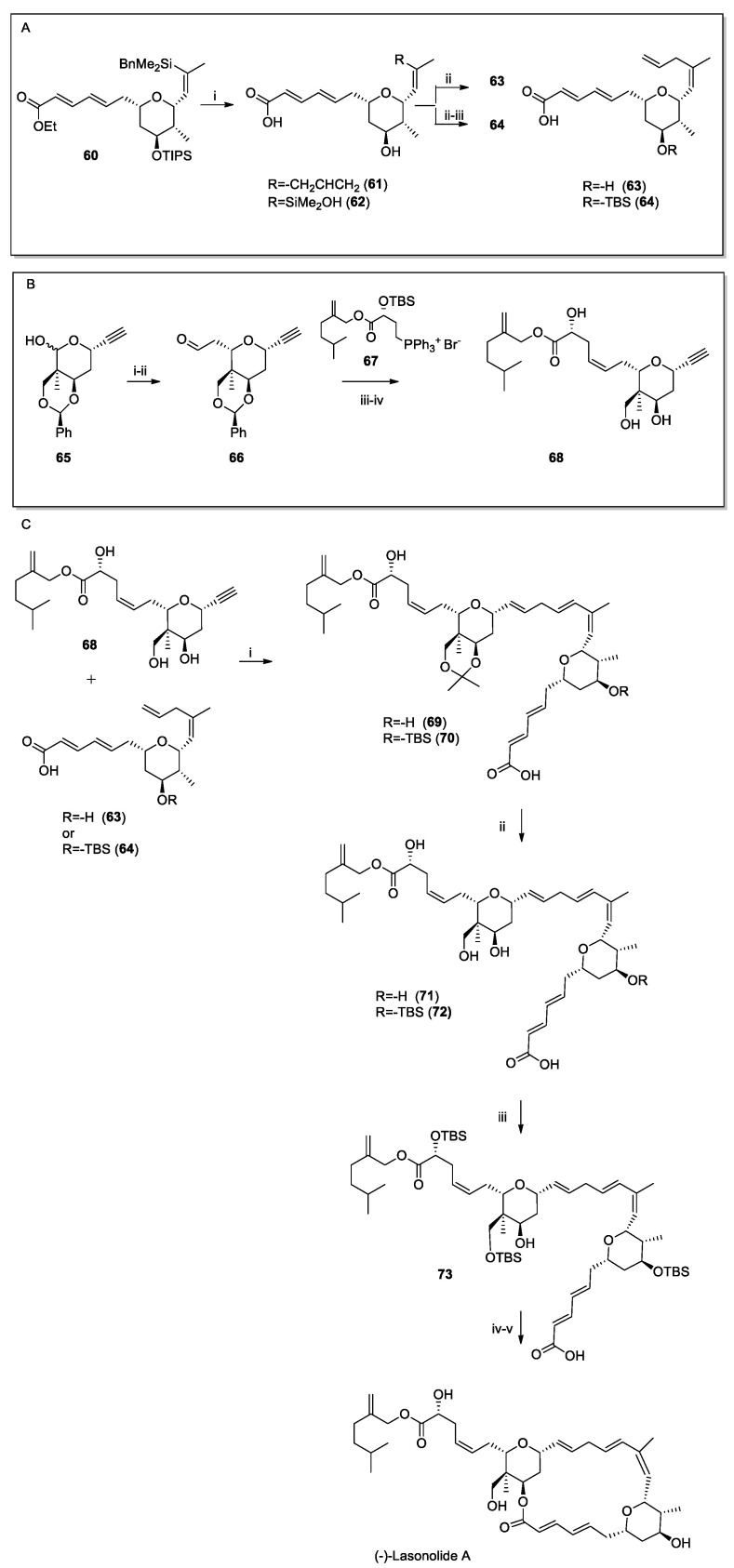
(**A**) Synthesis of compound **64** [89]. Reagent and conditions: (i) Pd_2_(dba)_3_·CHCl_3_, allyl acetate, TBAF; (ii) LiOH; (iii) TBSOTf, 2,6-luditine. (**B**) Synthesis of Compound **68** [89]. Reagent and conditions: (i) Tert-butyl 2-(diethoxyphosphoryl)acetate, NaH, THF; (ii) DIBAL-H, CH_2_Cl_2_; (iii) **67**, KHMDS, THF; (iv) LiBF_4_, aq. MeCN. (**C**) Synthesis of (−)-Lasonolide A [89]. Reagent and conditions: (i) [CpRu(MeCN)_3_]PF_6_, (ii) CSA; (iii) TBSCl, ImH, 1M HCl; (iv) 2,4,6-trichlorobenzoyl chloride, Et_3_N, DMAP; (v) HF·Pyr.

In 2016, Trost et al. [89] accomplished the last total synthesis of (−)-lasonolide A starting from two key intermediates synthesised by the same group (compounds **64** and **67**, Scheme 9A,B). The coupling between these two compounds was performed and optimised by using Ru-catalysed coupling (Scheme 9C) [90]. Both intermediates **64** and **68** were used for the reaction of acetonide with CSA to obtain both **69** and **70**. Then, the protection of the most accessible alcohols in TMSCl, ImH, 1M HCl, followed by macrolactonization with the Yamaguchi reagent, produced the TBS-protected (−)-lasonolide A.

### 2.4. Peptides: Linear, Cyclic, and Depsipeptides

Dolastatins, diazonamide A, thiocoraline, and vitilevuamide (Figure 5 and Table 4) are among the marine-derived natural peptides that have garnered considerable attention due to their potent anticancer activity. These compounds display unique structural features and mechanisms of action, making them valuable leads for the development of novel chemotherapeutic agents. However, the total syntheses of these molecules were accomplished years ago, and no recent synthetic advancements have been reported, reflecting both the complexity of their structures and the challenges associated with their large-scale production.

Dolastatins (Figure 5 and Table 4) and a few other related substances are antineoplastic pseudopeptides that were extracted from the nudibranch *Dolabella auricularia* [91,92]. Dolastatin 10 and 15 exhibited the most pronounced antiproliferative effect among all the metabolites and are currently being assessed for clinical studies [91]. The stereochemical features and structure of dolastatin-16 were clarified by X-ray crystallography in 2011. Surprisingly, unlike the natural extract, a later complete synthesis produced a molecule that was spectroscopically equivalent to the natural product but lacked nanomolar anticancer action in vitro [93]. It has been suggested that the bioactivity that was previously ascribed to the natural extract might have been caused by certain conformations that were missing from the synthesised molecule or by unknown impurities [93]. As a result, there are still many questions regarding dolastatin 16’s actual anticancer potential. Due to the lack of activity seen in its synthetic form, dolastatin 16 never reached the clinical stage, unlike dolastatins 10 and 15, which advanced to phase I–III clinical trials as already reported above.

On the other hand, the ascidians *Diazona angulate* are the source of a complex of the macrocyclic peptides named diazonamides [94]. The anticancer properties of diazonamide A (Figure 5 and Table 4) were assessed among other diazolamides [94]. It binds tubulin and stops the cell cycle during the G2/M phase. In animal models, diazonamide A is a potential chemotherapeutic drug that does not cause severe toxicity [95]. Nicolaou’s group accomplished the first complete synthesis of diazonamide A in 2002 [96]. This work is very significant since it combines Pd-boryl annulation for the second ring, Mg^2+^-assisted macro-aldolization with DAST for the first ring, and asymmetric catalysis mediated by the iminium ion to create a complex macrocycle. Despite having an overall yield of less than 2% and being finished in 20 linear steps, the total synthesis is particularly noteworthy in the field of total synthesis. In order to avoid the macro-aldolizations employed in the first procedure, the second synthesis of diazonamide, carried out by the same group [97], adopts a totally new approach, using SmI_2_ for a ring-closing sequence and a unique Pd-mediated oxidation to transform an indoline into an oxindole. Furthermore, MacMillan group total synthesis of diazonamide A [98] (2010) involved an iminium-catalysed stereoselective cascade to construct the furanoindoline core and C(10) quaternary centre. Macrocycle formation depended on Pd-catalysed borylation/annulation and Mg^2+^-mediated aldolization, completing the synthesis in 20 steps with an overall yield of 1.8%.

The marine bacterium *Micromonospora marina* is the source of the cyclic depsipeptide thiocoraline (Figure 5 and Table 4) [99]. Thiocoraline does not inhibit DNA topoisomerase II or cause DNA strand breaks. Instead, its anticancer activity is linked to inhibition of DNA replication [100]. Primer extension assays showed that thiocoraline blocks DNA elongation by DNA polymerase α at concentrations that also halt cell-cycle progression and colony formation, indicating that the inhibition of DNA polymerase α activity is the primary mechanism [100]. Strong cytotoxicity is demonstrated by thiocoraline against a variety of cancer cell lines, especially colon cancer cell lines and those that are resistant to drugs [101]. Thiocoraline has not yet progressed to clinical trials, despite showing promise in preclinical research. A disulfide bridge connects a symmetrical dimer in its complicated structure. Because of its intricate symmetrical bicyclic depsipeptide structure, which consists of two identical peptide chains connected by a disulfide bridge, synthetic chemists have found it difficult to synthesise thiocoraline in its entirety. In the early 2000s, Boger and colleagues accomplished the first total synthesis [102]. Prior to dimerisation and the creation of disulfide bonds, they assembled each half of the molecule independently using a convergent strategy. This entire synthesis provides access to synthetic analogues that enhance pharmacological properties and enable thorough biological assessments [103].

Vitilevuamide (Figure 5 and Table 4) is a bicyclic peptide that was extracted from marine ascidians and exhibits strong cytotoxicity against a variety of tumour cell lines (LC_50_: 6–311 nM) and acts as a tubulin polymerisation inhibitor, displaying potency comparable to colchicine [104]. In vitro, vitilevuamide inhibits tubulin assembly (IC_50_ ≈ 2 µM) and causes G_2_/M cell-cycle arrest with 78% of cells becoming tetraploid. It also noncompetitively inhibits vinblastine binding and stabilises colchicine binding, suggesting interaction at a distinct tubulin site. In vivo, it significantly prolonged the survival of mice with P388 leukaemia by 70% at 30 µg/kg, confirming its antimitotic and antitumor activity [104].

Vitilevuamide has not yet been completely synthesised and published in peer-reviewed literature. The majority of the published data focuses on the compound’s structural characterisation by NMR and MS/MS, as well as its isolation from the ascidians *Didemnum cuculiferum* and *Polysyncraton lithostrotum*. Its potent in vitro and in vivo tubulin-inhibiting action is one of the biological data; nevertheless, no comprehensive synthesis pathway has been revealed thus far.

**Table 4:** Marine-derived peptides and depsipeptides with anticancer activities: chemical classification, source, and involvement in cancer.

Compound (Isolation)	Class	Source	Cancer Type
Dolastatin 15 (1987)	Linear peptide	*Dolabella auricularia* (mollusc)	Lymphoma, breast cancer
Diazonamide (1994)	Cyclic peptide	*Diazona angulata* (tunicate)	Colon, pancreatic cancers
Thiocoraline (1999)	Depsipeptide	*Micromonospora marina* (bacterium)	Neuroblastoma, lung cancer
Vitilevuamide (2002)	Cyclic peptide	*Didemnuin cuculiferum* (tunicates)	Lung, breast cancers

### 2.5. Other Classes

Further marine-derived compounds belong to different chemical classes. In this paragraph, we will describe curacin A, spisulosin 1, and dictyodendrins A–E as the only known members of these classes able to exhibit notable antitumor activity.

A naturally occurring substance with strong anticancer effects, curacin A is a thiazole lipid (Figure 6 and Table 5) and it was extracted from the marine cyanobacterium *Lyngbya majuscula*. Curacin A attaches itself to β-tubulin’s colchicine-binding site. Since microtubules are necessary for the creation of mitotic spindles, their formation is inhibited [105]. Consequently, apoptosis results from the arrest of cancer cells in the G2/M phase of the cell cycle.

In vitro, curacin A has shown strong antiproliferative actions against HT-29, MCF-7, and A549 [105]. The clinical development of curacin A is severely hampered by its low water solubility and lack of chemical stability; in light of this, analogues and derivatives have been investigated for increased stability and bioavailability for in vivo applications, although they have been mostly examined in vitro [105].

On the other hand, originally obtained from the arctic surf clam *Spisula polynyma*, spisulosine 1 (Figure 6 and Table 5) is a marine-derived aminodiol molecule that shares structural similarities with sphingosine. When spisulosine 1 was initially isolated from the clam *Spisula polynyma* (syn. *Mactrometris polynyma*), it exhibited a high IC_50_ for cytotoxic activity against both solid tumour cells and leukaemia cell lines [106,107,108].

Padrón and colleagues further demonstrated that this compound has extraordinary antiproliferative efficacy on a number of cancer cells (HBL-100, HeLa, SW1573, T-47D, and WiDr) and acts as a specific inhibitor of casein kinase 1ε (CK1ε). In human pulmonary artery smooth muscle cells (PASMCs), Bittman and colleagues discovered that spisulosine specifically inhibits sphingosine kinase (SphK1) to cause its ubiquitin-proteasomal degradation, with an IC_50_ of 7.1 ± 0.75 mM, and also considerably suppresses the production of DNA in PASMC. Fusetani and Matsunaga identified dictyodendrins A−E (Figure 6 and Table 5) from the *Dictyodendrilla verongiformis* sponge collected off the coast of southern Japan [109]. These substances are members of a novel class of alkaloids that have at least one sulphate group on the periphery and a distinctive pyrrolo[2,3-c]carbazole moiety. Telomerase inhibition is a prospective target for cancer chemotherapy since the telomerase enzyme is overexpressed in >85% of tumour cells but not in normal cells [110]. Due to their distinct chemical structures and encouraging biological activities, numerous chemists have studied the complete synthesis of dictyodendrins [111,112,113,114,115,116,117,118,119].

**Table 5:** Curacin A, spisulosine, dictyodendrins: chemical classification, source, and involvement in cancer.

Compound (Isolation)	Class	Source	Cancer Type
Curacin A (1994)	Thiazole lipid	*Lyngbya majuscula* (cyanobacterium)	Colon cancer
Spisulosine (ES-285) (2000)	Alkylamino alcohol	*Mactromeris polynyma* (mollusc)	Breast cancer
Dictyodendrins (1993–2012)	Pyrrolocarbazole derivatives	*Dictyodendrilla verongiformis* (sponge)	Melanoma, glioblastoma

#### Recent Total Synthesis of Spisulosine and Dictyodendrin B

In 2010, Arun K. Shaw and Partha Ghosal [120] achieved the total synthesis of spisulosine. They first prepared (*S*)-Garner’s aldehyde in nine steps. To establish the desired 2*S*,3*R* configuration, a key step involved a diastereoselective vinyl Grignard addition to Garner’s aldehyde. The long C_18_ alkyl chain was introduced via olefin cross-metathesis, and final hydrogenation afforded (+)-spisulosine (ES 285). Since then, the total synthesis of this natural compound has also been reported by other groups [121,122].

**Scheme 10:**
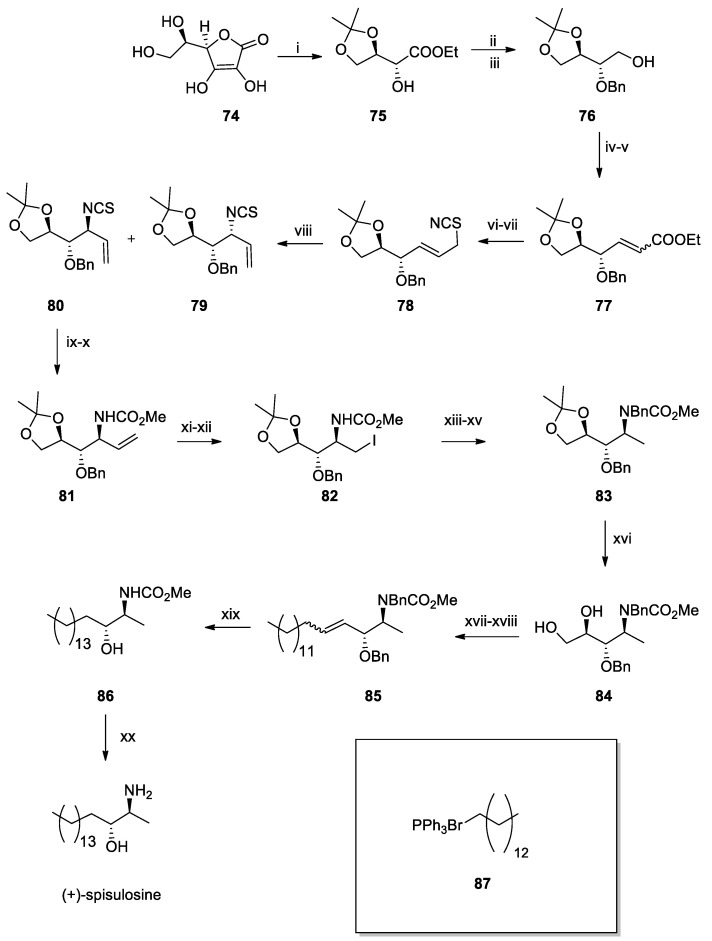
Synthesis of (+)-spisulosine reported by Göngiovà group [123]. Reagents and conditions: (i) Ref. [124]; (ii) BnBr, Ag_2_O, CH_2_Cl_2_, rt; (iii) LiAlH_4_, THF, rt; (iv) IBX, MeCN, reflux; (v) Ph_3_P = CHCO_2_Et, CH_2_Cl_2_, rt; (vi) DIBAl-H, CH_2_Cl_2_, −50 °C; (vii) MsCl, Et_3_N, CH_2_Cl_2_, 0 °C to r.t.; (viii) KSCN, MeCN, 0 °C to r.t.; (viii) heptane, MW, 70 °C, 1 h or Δ, 90 °C, 1 h; (ix) MeONa, MeOH, 0 °C to r.t.; (x) mesitylnitrile oxide (MNO), MeCN, rt; (xi) O_3_, MeOH/CH_2_Cl_2_, −78 °C; (xii) NaBH_4_, −78 °C to 0 °C; (xiii) Ph_3_P, I_2_, imidazole, Et_2_O/MeCN, r.t.; (xiv) H_2_, 10% Pd/C, MeOH, Et_3_N, r.t.; (xv) BnBr, NaH, TBAI, DMF, 0 °C to r.t.; (xvi) p-TsOH, MeOH, r.t.; (xvii) NaIO_4_, MeOH/H_2_O (1:1), r.t.; (xviii) LHMDS, THF, **87**, r.t.; (xix) H_2_, 10% Pd/C/20% Pd(OH)_2_/C (1:1), MeOH, 60 °C; (xx) 2M aq NaOH, EtOH, reflux.

In 2016, Göngiovà group [123] reported the synthesis of (+)-spisulosine (Scheme 10) and its anticancer activity, which shows an IC_50_ value < 1 µM on different cancer cell lines. Regarding the synthesis, the authors started from D-isoascorbic acid (**74**), which was converted into intermediate **75**, as previously reported by Abushanab et al. [124]. Compound **75** was then subjected to benzylation of the free hydroxy group using BnBr and Ag_2_O in CH_2_Cl_2_, followed by reduction of the ester function to yield compound **76**. A one-pot IBX oxidation/Wittig olefination of compound **76** produced a mixture of α,β-unsaturated esters **77**, with the *E* isomer predominating over the *Z* (ratio 88:12). After chromatographic separation, the major *E*-ester was reduced using DIBAL-H, affording the required allylic alcohol. Subsequent reaction with MsCl and Et_3_N, followed by treatment with KSCN in MeCN, yielded the corresponding isothiocyanate in two diastereomeric forms (compounds **79** and **80**). This step can be performed under different conditions: microwave irradiation at 70 °C for 1 h yields a 78:22 ratio of the two diastereomers, and similar results are obtained under conventional heating at 90 °C for 1 h. The authors observed that increasing the reaction time and temperature favoured the formation of compound **79**, likely due to the higher stability of this diastereomer compared to the other.

Isothiocyanate **80** was transformed into carbamate **81** over two steps: reaction with MeONa, followed by treatment with mesityl nitrile oxide (MNO). Ozonolysis of compound **81**, followed by a reductive treatment with NaBH_4_, furnished compound **82**. Following the procedure described by Garegg’s group, alcohol **82** was converted into iodide, which underwent catalytic hydrogenation (10% Pd/C) in the presence of Et_3_N. To overcome the low yield in this transformation, the carbamate nitrogen was protected with a benzyl group, yielding the desired derivative **83**. Subsequent p-toluenesulfonic acid-mediated acetonide hydrolysis afforded compound **84**. This functionalized intermediate was subjected to oxidative fragmentation with NaIO_4_ to give the corresponding aldehyde, which was treated with LHMDS and compound **87** in THF, resulting in a poorly separable mixture of olefins **85**. Catalytic hydrogenation saturated the double bond and removed both benzyl ether protecting groups, converting compound **85** into **86**. Finally, base hydrolysis (2 M aqueous NaOH in EtOH, reflux) provided the target compound, (+)-spisulosine.

**Scheme 11:**
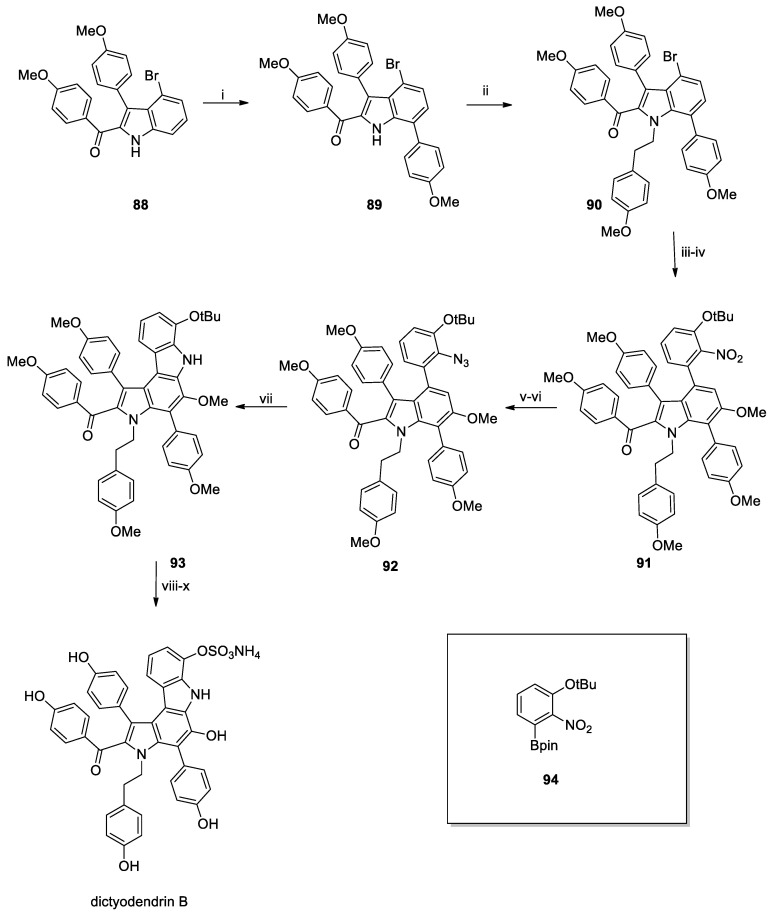
Synthesis of dictyodendrin B reported by M. J. Gaunt [125]. Reagent and conditions: (i) (Bpin)_2_, [{IrCl(cod)}leo_2_], 4,4′-di-tert-butyl-2,2′-dipyridyl, THF, 90 °C, 1.5 h; 4-iodoanisole, [PdCl_2_(dppf)], KOH, 80 °C, 30 min; (ii) 4-methoxyphenethylbromide, K_2_CO_3_, DMF, 100 °C, 16 h; (iii) **94**, [PdCl_2_(dppf)], K_2_CO_3_, dioxane, 90 °C, 20 h; (iv) NBS, DMF, rt, 24 h, NaOMe, CuI, 80 °C 18.5 h; (v) Pd(OH)_2_, H_2_, MeCN, 24 h; (vi) AcOH, tBuONO, TMSN_3_, RT, 20 min; (vii) dioxane, 180 °C, 30 min residence time, 0.333 mL min, Vapourtec R2+/R4 system; (viii-x) see Ref. [111].

Regarding the dictyodendrin B, in 2015, Gaunt [125] performed an efficient and innovative approach to synthesise it (Scheme 11), starting from a simple and commercially available indole building block. One of the main strengths of the synthesis is the use of catalytic C–H functionalization reactions and highly selective electrophilic aromatic substitutions, even in complex molecular environments. Another notable aspect is the use of flow chemistry for the late-stage formation of the carbazole ring, which adds both precision and scalability to the process. Overall, the method is modular and streamlined, allowing for the production of over a gram of the protected natural product and paving the way for the creation and testing of various analogues with potentially enhanced biological activity. In more detail, the indole’s C7 position is targeted for iridium-catalysed C–H borylation, taking advantage of the directing ability of the indole nitrogen. This borylated intermediate is then directly subjected to a Suzuki–Miyaura coupling with 4-iodoanisole, installing an aryl group at C7 and yielding compound **88**. With the C7 arylation complete, attention turns to the N1 position, which is alkylated using 4-methoxyphenethyl bromide under basic conditions. The resulting product compound **90** is then subjected to a second Suzuki coupling at the C4 position, using a specially prepared nitrophenol-derived boronic ester. This step introduces the final aryl ring. The newly installed nitrophenyl group allows for selective bromination at C6 using N-bromosuccinimide (NBS). Directly in the same pot, the resulting aryl bromide is converted into a methoxy group via copper-catalysed substitution, producing compound **91**. The nitro group is then transformed into an azide through a three-step sequence: reduction, diazotisation, and azidation, yielding compound **92**. This azide is a key precursor for forming the central carbazole ring of dictyodendrin B, applying a continuous-flow process to thermally decompose the azide. This generates a nitrene intermediate that undergoes intramolecular C–H amination, forming the fused carbazole core in compound **93**. At this point, the final stages involve the removal of the tert-butyl ether and sulfonylation of the newly formed hydroxy group, demethylation of the other hydroxy group, and finally, reductive desulfonylation. These transformations culminate in the formation of dictyodendrin B, whose structure matches the natural product in all respects.

**Scheme 12:**
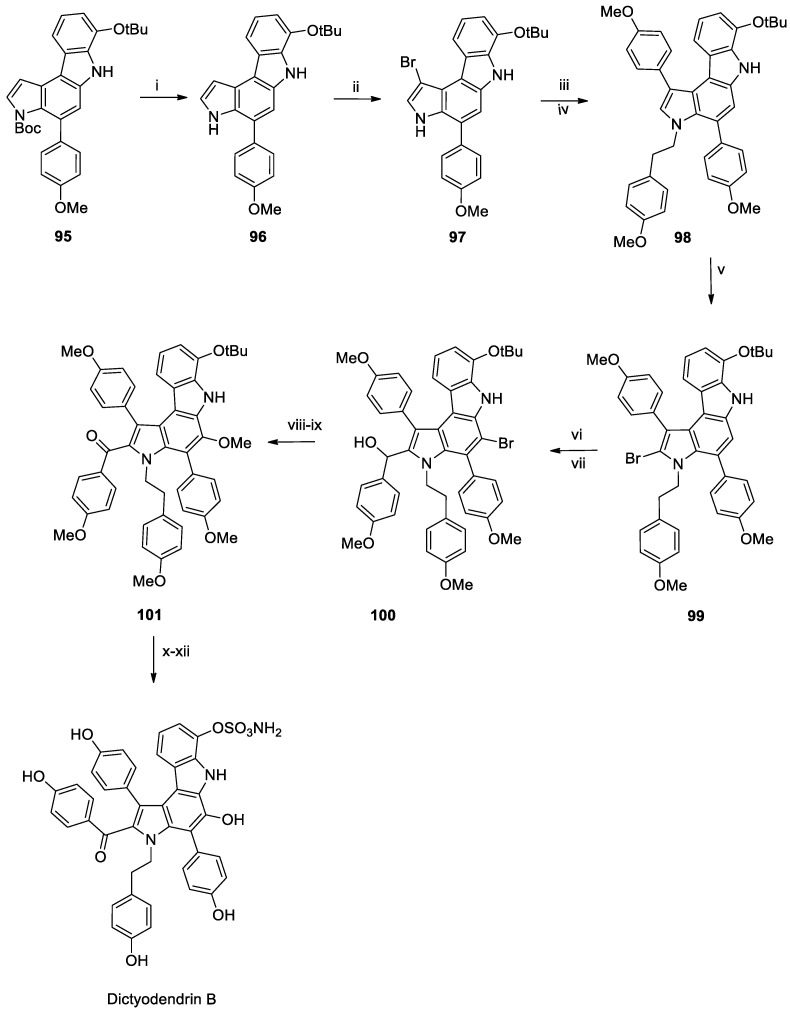
Synthesis of dictyodendrin B reported by Ohno’s group [126]. Reagent and conditions: (i) NaOMe, THF; (ii) NBS, THF; (iii) 1-(2-bromoethyl)-4-methoxybenzene, NaOH, 18-C-6, H_2_O; (iv) (4-methoxyphenyl)boronic acid, [Pd(tBu_3_P)_2_], K_3_PO_4_, dioxane/H_2_O; (v) NBS, THF; (vi) MeLi, nBuLi, p-anisaldehyde; (vii) NBS, THF; (viii) [TRAP]NMO, CH_2_Cl_2_; (ix) CuI, NaOMe, DMF; (x) BCl_3_, C_6_HMe_5_, CH_2_Cl_2_; (xi) CCl_3_CH_2_OSO_2_Cl, DABCO, CH_2_Cl_2_; (xii) BCl_3_, nBuNI, CH_2_Cl_2_; (xiii) Zn dust, HCO_2_NH_4_, MeOH.

In 2020, Ohno’s group [126] reported the total synthesis of dictyodendrin B (Scheme 12) and the formal synthesis of the other dictyodendrins. Starting from compound **95**, the Boc group was first removed with NaOMe to increase the bromination in NBS in THF. In the next step, the alkylation of the nitrogen of the indole ring was obtained with NaOH, 18-crown-6, and H_2_O. Finally, the Suzuki cross-coupling with 4-methoxyphenyl boronic acid, [Pd(tBu_3_P)_2_], K_3_PO_4_, dioxane/H_2_O gave compound **94**. This was selectively brominated, which undergoes Ley–Griffith oxidation. Ullmann coupling with NaOMe was used to introduce a methoxy group at the C5 position, affording compound **97**. 

Compound **97** was then subjected to the removal of the OtBu group with BCl_3_ at −78 °C, formation of the sulphate group (following Tokuyama’s protocol) and removal of the remaining protecting groups (BCl_3_ + Zn), to yield dictyodendrin B.

## 3. Conclusions

Marine organisms, such as sponges, algae, and tunicates, have yielded a variety of secondary metabolites over the past few decades, including peptides, alkaloids, macrocyclic lactones, and terpenes. Some of these compounds have successfully advanced to the pharmaceutical market, while many others are progressing through preclinical and early clinical evaluation, particularly as candidates for cancer therapy. This review highlights the subset of marine natural products that are currently being studied for their anticancer properties, with a particular focus on how total synthesis has contributed to their production. Drug development and widespread use are severely hampered by the structural complexity and paucity of these metabolites from natural sources. Therefore, chemical synthesis and/or semi-synthetic approaches are necessary to ensure long-term access and facilitate structure–activity connection research. Specifically, we focus our attention on the more recent total synthesis, with a particular emphasis on the past decade.

Aromatic and heterocyclic alkaloids, terpenes and their derivatives, macrolides and macrocycles, linear, cyclic, or depsipeptide peptides, and other diverse frameworks such as polyketides, thiazole lipids, alkylamino alcohols, and pyrrolocarbazole derivatives are the main classes into which the compounds discussed here can be broadly divided. This work demonstrates the remarkable potential of marine natural products for cancer medication development and encourages further.

## Data Availability

No new data were created or analyzed in this study. Data sharing is not applicable to this article.

## References

[1] Altmann K.-H. (2017). Drugs from the oceans: Marine natural products as leads for drug discovery. Chimia.

[2] Perry N.B., Ettouati L., Litaudon M., Blunt J.W., Munro M.H., Parkin S., Hope H. (1994). Alkaloids from the antarctic sponge Kirkpatrickia varialosa.: Part 1: Variolin b, a new antitumour and antiviral compound. Tetrahedron.

[3] Lu W.-Y., Li H.-J., Li Q.-Y., Wu Y.-C. (2021). Application of marine natural products in drug research. Biorg. Med. Chem..

[4] Mayer A.M.S., Glaser K.B., Cuevas C., Jacobs R.S., Kem W., Little R.D., McIntosh J.M., Newman D.J., Potts B.C., Shuster D.E. (2010). The odyssey of marine pharmaceuticals: A current pipeline perspective. Trends Pharmacol. Sci..

[5] Bergmann W., Feeney R.J. (1951). Contributions to the study of marine products. XXXII. The nucleosides of sponges. I. J. Org. Chem..

[6] Löwenberg B., Pabst T., Vellenga E., van Putten W., Schouten H.C., Graux C., Ferrant A., Sonneveld P., Biemond B.J., Gratwohl A. (2011). Cytarabine dose for acute myeloid leukemia. N. Engl. J. Med..

[7] Murphy T., Yee K.W. (2017). Cytarabine and daunorubicin for the treatment of acute myeloid leukemia. Expert Opin. Pharmacother..

[8] Chhikara B.S., Parang K. (2010). Development of cytarabine prodrugs and delivery systems for leukemia treatment. Expert Opin. Drug Deliv..

[9] Moser A.M., Adamson P.C., Gillespie A.J., Poplack D.G., Balis F.M. (1999). Intraventricular concentration times time (C × T) methotrexate and cytarabine for patients with recurrent meningeal leukemia and lymphoma. Cancer.

[10] Ganjoo K.N., Patel S. (2009). Trabectedin: An anticancer drug from the sea. Expert Opin. Pharmacother..

[11] Huryn D., Wipf P. (2014). Natural product chemistry and cancer drug discovery. Cancer Drug Des. Discov..

[12] Pettit G.R., Kamano Y., Herald C.L., Tuinman A.A., Boettner F.E., Kizu H., Schmidt J.M., Baczynskyj L., Tomer K.B., Bontems R.J. (1987). The isolation and structure of a remarkable marine animal antineoplastic constituent: Dolastatin 10. J. Am. Chem. Soc..

[13] Turner T., Jackson W.H., Pettit G.R., Wells A., Kraft A.S. (1998). Treatment of human prostate cancer cells with dolastatin 10, a peptide isolated from a marine shell-less mollusc. Prostate.

[14] Prabhu R.H., Patravale V.B. (2020). Marine-derived pharmaceuticals for oncotherapy: Clinical trial and FDA-approved compounds. Encyclopedia of Marine Biotechnology.

[15] Kobayash J.I., Cheng J.-F., Nakamura H., Ohizumi Y., Hirata Y., Sasaki T., Ohta T., Nozoe S. (1988). Ascididemin, a novel pentacyclic aromatic alkaloid with potent antileukemic activity from the okinawan tunicate didemnum sp. Tetrahedron Lett..

[16] Dassonneville L., Wattez N., Baldeyrou B., Mahieu C., Lansiaux A., Banaigs B., Bonnard I., Bailly C. (2000). Inhibition of topoisomerase II by the marine alkaloid ascididemin and induction of apoptosis in leukemia cells. Biochem. Pharmacol..

[17] Bonnard I., Bontemps N., Lahmy S., Banaigs B., Combaut G., Francisco C., Colson P., Houssier C., Waring M., Bailly C. (1995). Binding to DNA and cytotoxic evaluation of ascididemin, the major alkaloid from the Mediterranean ascidian Cystodytes dellechiajei. Anti-Cancer Drug Des..

[18] Dirsch V.M., Kirschke S.O., Estermeier M., Steffan B., Vollmar A.M. (2004). Apoptosis signaling triggered by the marine alkaloid ascididemin is routed via caspase-2 and JNK to mitochondria. Oncogene.

[19] Duc D.X., Quoc N.V. (2022). Isolation, Bioactivities, and Synthesis of Lamellarin Alkaloids: A Review. Curr. Org. Chem..

[20] Dias N., Vezin H., Lansiaux A., Bailly C. (2005). Topoisomerase inhibitors of marine origin and their potential use as anticancer agents. DNA Binders and Related Subjects.

[21] Chittchang M., Batsomboon P., Ruchirawat S., Ploypradith P. (2009). Cytotoxicities and structure–activity relationships of natural and unnatural lamellarins toward cancer cell lines. ChemMedChem.

[22] Ballot C., Kluza J., Lancel S., Martoriati A., Hassoun S.M., Mortier L., Vienne J.-C., Briand G., Formstecher P., Bailly C. (2010). Inhibition of mitochondrial respiration mediates apoptosis induced by the anti-tumoral alkaloid lamellarin D. Apoptosis.

[23] Dembitsky V.M., Gloriozova T.A., Poroikov V.V. (2005). Novel antitumor agents: Marine sponge alkaloids, their synthetic analogs and derivatives. Mini Rev. Med. Chem..

[24] Anderson R.J., Hill J.B., Morris J.C. (2005). Concise total syntheses of variolin B and deoxyvariolin B. J. Org. Chem..

[25] Walker S.R., Carter E.J., Huff B.C., Morris J.C. (2009). Variolins and related alkaloids. Chem. Rev..

[26] Simone M., Erba E., Damia G., Vikhanskaya F., Di Francesco A.M., Riccardi R., Bailly C., Cuevas C., Sousa-Faro J.M.F., D’Incalci M. (2005). Variolin B and its derivate deoxy-variolin B: New marine natural compounds with cyclin-dependent kinase inhibitor activity. Eur. J. Cancer.

[27] Bettayeb K., Tirado O.M., Marionneau-Lambot S., Ferandin Y., Lozach O., Morris J.C., Mateo-Lozano S., Drueckes P., Schächtele C., Kubbutat M.H. (2007). Meriolins, a new class of cell death–inducing kinase inhibitors with enhanced selectivity for cyclin-dependent kinases. Cancer Res..

[28] Imperatore C., Aiello A., D’Aniello F., Senese M., Menna M. (2014). Alkaloids from marine invertebrates as important leads for anticancer drugs discovery and development. Molecules.

[29] Baeza A., Mendiola J., Burgos C., Alvarez-Builla J., Vaquero J. (2008). Palladium-mediated C–N, C–C, and C–O functionalization of azolopyrimidines: A new total synthesis of Variolin B. Tetrahedron Lett..

[30] Baeza A., Mendiola J., Burgos C., Alvarez-Builla J., Vaquero J.J. (2010). Application of Selective Palladium-Mediated Functionalization of the Pyrido [3′,2′:4,5] pyrrolo [1,2-c] pyrimidine Heterocyclic System for the Total Synthesis of Variolin B and Deoxyvariolin B. EurJoc.

[31] Ishibashi F., Miyazaki Y., Iwao M. (1997). Total syntheses of lamellarin D and H. The first synthesis of lamellarin-class marine alkaloids. Tetrahedron.

[32] Komatsubara M., Umeki T., Fukuda T., Iwao M. (2014). Modular synthesis of lamellarins via regioselective assembly of 3,4,5-differentially arylated pyrrole-2-carboxylates. J. Org. Chem..

[33] Li Q., Jiang J., Fan A., Cui Y., Jia Y. (2011). Total synthesis of lamellarins D, H, and R and ningalin B. Org. Lett..

[34] Handy S.T., Zhang Y. (2005). Approaches to the synthesis of the lamellarins and related natural products. A review. Org. Prep. Proced. Int..

[35] Imbri D., Tauber J., Opatz T. (2014). Synthetic approaches to the lamellarins—A comprehensive review. Mar. Drugs.

[36] Sivaganesan P., VL S., Sahoo A., Elanchezhian C., Nataraj G., Chaudhuri S. (2024). A Comprehensive Review of Synthetic Approaches Toward Lamellarin D and its Analogous. ChemistrySelect.

[37] Theppawong A., Ploypradith P., Chuawong P., Ruchirawat S., Chittchang M. (2015). Facile and divergent synthesis of lamellarins and lactam-containing derivatives with improved drug likeness and biological activities. Chem. Asian J..

[38] Manjappa K.B., Syu J.-R., Yang D.-Y. (2016). Visible-light-promoted and Yb (OTf) 3-catalyzed constructions of coumarin-pyrrole-(iso) quinoline-fused pentacycles: Synthesis of lamellarin core, lamellarin D trimethyl ether, and lamellarin H. Org. Lett..

[39] Manjappa K.B., Lin J.-M., Yang D.-Y. (2017). Construction of pentacyclic lamellarin skeleton via Grob reaction: Application to total synthesis of lamellarins H and D. J. Org. Chem..

[40] Lade D.M., Pawar A.B., Mainkar P.S., Chandrasekhar S. (2017). Total synthesis of lamellarin D trimethyl ether, lamellarin D, and lamellarin H. J. Org. Chem..

[41] Mei R., Zhang S.-K., Ackermann L. (2017). Concise Synthesis of Lamellarin Alkaloids by C–H/N–H Activation: Evaluation of Metal Catalysts in Oxidative Alkyne Annulation. Synlett.

[42] Shirley H.J., Koyioni M., Muncan F., Donohoe T.J. (2019). Synthesis of lamellarin alkaloids using orthoester-masked α-keto acids. Chem. Sci..

[43] Liang L.F., Guo Y.W. (2013). Terpenes from the soft corals of the genus Sarcophyton: Chemistry and biological activities. Chem. Biodivers..

[44] Hamel E., Sackett D.L., Vourloumis D., Nicolaou K. (1999). The coral-derived natural products eleutherobin and sarcodictyins A and B: Effects on the assembly of purified tubulin with and without microtubule-associated proteins and binding at the polymer taxoid site. Biochemistry.

[45] Pietra F. (2020). Fighting cancer with microtubule-stabilizing agents: A computational investigation of the complex between β-tubulin and the microtubule-stabilizing, antitumor marine diterpenoid sarcodictyin A. Struct. Chem..

[46] Risinger A.L., Giles F.J., Mooberry S.L. (2009). Microtubule dynamics as a target in oncology. Cancer Treat. Rev..

[47] Nicolaou K., Pfefferkorn J., Xu J., Winssinger N., Ohshima T., Kim S., Hosokawa S., Vourloumis D., Van Delft F., Li T. (1999). Total synthesis and chemical biology of the sarcodictyins. Chem. Pharm. Bull..

[48] Lindel T., Jensen P.R., Fenical W., Long B.H., Casazza A.M., Carboni J., Fairchild C.R. (1997). Eleutherobin, a new cytotoxin that mimics paclitaxel (Taxol) by stabilizing microtubules. J. Am. Chem. Soc..

[49] Sosonyuk S.E., Peshich A., Tutushkina A.V., Khlevin D.A., Lozinskaya N.A., Gracheva Y.A., Glazunova V.A., Osolodkin D.I., Semenova M.N., Semenov V.V. (2019). Synthesis and cytotoxicity of novel simplified eleutherobin analogues as potential antitumour agents. Org. Biomol. Chem..

[50] Su J.-H., Tseng S.-W., Lu M.-C., Liu L.-L., Chou Y., Sung P.-J. (2011). Cytotoxic C21 and C22 terpenoid-derived metabolites from the sponge Ircinia sp. J. Nat. Prod..

[51] Nicolaou K., Winssinger N., Vourloumis D., Ohshima T., Kim S., Pfefferkorn J., Xu J.-Y., Li T. (1998). Solid and solution phase synthesis and biological evaluation of combinatorial sarcodictyin libraries. J. Am. Chem. Soc..

[52] Driedger D., Fers-Lidou A., Schroeder M., Elisia I., Krystal G., Britton R. (2025). An Expedient Synthesis of the Antimitotic Natural Products Sarcodictyin and Eleutherobin, and Carbohydrate Analogues. J. Am. Chem. Soc..

[53] Zhao H., McMillan A.J., Constantin T., Mykura R.C., Julia F., Leonori D. (2021). Merging halogen-atom transfer (XAT) and cobalt catalysis to override E2-selectivity in the elimination of alkyl halides: A mild route toward contra-thermodynamic olefins. J. Am. Chem. Soc..

[54] Nicolaou K., Van Delft F., Ohshima T., Vourloumis D., Xu J., Hosokawa S., Pfefferkorn J., Kim S., Li T. (1997). Total synthesis of eleutherobin. Angew. Chem. Int. Ed. Engl..

[55] Nicolaou K., Xu J.-Y., Kim S., Ohshima T., Hosokawa S., Pfefferkorn J. (1997). Synthesis of the tricyclic core of eleutherobin and sarcodictyins and total synthesis of sarcodictyin A. J. Am. Chem. Soc..

[56] Chen X.-T., Bhattacharya S.K., Zhou B., Gutteridge C.E., Pettus T.R., Danishefsky S.J. (1999). The total synthesis of eleutherobin. J. Am. Chem. Soc..

[57] Mowat J.S. (2012). Studies Towards the Total Synthesis of Eleutherobin and Other Marine Natural Products. Ph.D. Thesis.

[58] Carter R., Hodgetts K., McKenna J., Magnus P., Wren S. (2000). Studies on the stereoselective synthesis of the marine antitumor agent eleutherobin. Tetrahedron.

[59] Hickford S.J., Blunt J.W., Munro M.H. (2009). Antitumour polyether macrolides: Four new halichondrins from the New Zealand deep-water marine sponge *Lissodendoryx* sp. Biorg. Med. Chem..

[60] Dissanayake D.S., Nagahawatta D.P., Lee J.-S., Jeon Y.-J. (2024). Immunomodulatory effects of halichondrin isolated from marine sponges and its synthetic analogs in oncological applications. Mar. Drugs.

[61] Newman D.J. (2023). Drug discovery from natural sources. Curr. Pharmacol. Rep..

[62] Corley D.G., Herb R., Moore R.E., Scheuer P.J., Paul V.J. (1988). Laulimalides. New potent cytotoxic macrolides from a marine sponge and a nudibranch predator. J. Org. Chem..

[63] Mooberry S.L., Tien G., Hernandez A.H., Plubrukarn A., Davidson B.S. (1999). Laulimalide and isolaulimalide, new paclitaxel-like microtubule-stabilizing agents. Cancer Res..

[64] Mooberry S.L., Randall-Hlubek D.A., Leal R.M., Hegde S.G., Hubbard R.D., Zhang L., Wender P.A. (2004). Microtubule-stabilizing agents based on designed laulimalide analogues. Proc. Natl. Acad. Sci. USA.

[65] Prota A.E., Bargsten K., Northcote P.T., Marsh M., Altmann K.H., Miller J.H., Díaz J.F., Steinmetz M.O. (2014). Structural basis of microtubule stabilization by laulimalide and peloruside A. Angew. Chem. Int. Ed..

[66] Horton P.A., Koehn F.E., Longley R.E., McConnell O.J. (1994). Lasonolide A, a new cytotoxic macrolide from the marine sponge *Forcepia* sp. J. Am. Chem. Soc..

[67] Isbrucker R.A., Guzman E.A., Pitts T.P., Wright A.E. (2009). Early effects of lasonolide A on pancreatic cancer cells. J. Pharmacol. Exp. Ther..

[68] Zhang Y.-W., Ghosh A.K., Pommier Y. (2012). Lasonolide A, a potent and reversible inducer of chromosome condensation. Cell Cycle.

[69] West L.M., Northcote P.T., Battershill C.N. (2000). Peloruside A: A potent cytotoxic macrolide isolated from the New Zealand marine sponge *Mycale* sp.. J. Org. Chem..

[70] Liao X., Wu Y., De Brabander J.K. (2003). Total synthesis and absolute configuration of the novel microtubule-stabilizing agent peloruside A. Angew. Chem..

[71] McGowan M.A., Stevenson C.P., Schiffler M.A., Jacobsen E.N. (2010). An enantioselective total synthesis of (+)-peloruside A. Angew. Chem..

[72] Jin M., Taylor R.E. (2005). Total synthesis of (+)-peloruside A. Org. Lett..

[73] Ghosh A.K., Xu X., Kim J.-H., Xu C.-X. (2008). Enantioselective total synthesis of peloruside A: A potent microtubule stabilizer. Org. Lett..

[74] Wilmes A., Bargh K., Kelly C., Northcote P.T., Miller J.H. (2007). Peloruside A synergizes with other microtubule stabilizing agents in cultured cancer cell lines. Mol. Pharm..

[75] Aicher T.D., Buszek K.R., Fang F.G., Forsyth C.J., Jung S.H., Kishi Y., Matelich M.C., Scola P.M., Spero D.M., Yoon S.K. (1992). Total synthesis of halichondrin B and norhalichondrin B. J. Am. Chem. Soc..

[76] Jackson K.L., Henderson J.A., Motoyoshi H., Phillips A.J. (2009). A total synthesis of norhalichondrin B. Angew. Chem. Int. Ed..

[77] Yamamoto A., Ueda A., Brémond P., Tiseni P.S., Kishi Y. (2012). Total synthesis of halichondrin C. J. Am. Chem. Soc..

[78] Yahata K., Ye N., Ai Y., Iso K., Kishi Y. (2017). Unified, Efficient, and Scalable Synthesis of Halichondrins: Zirconium/Nickel-Mediated One-Pot Ketone Synthesis as the Final Coupling Reaction. Angew. Chem..

[79] Ueda A., Yamamoto A., Kato D., Kishi Y. (2014). Total synthesis of halichondrin A, the missing member in the halichondrin class of natural products. J. Am. Chem. Soc..

[80] Wang Y., Habgood G.J., Christ W.J., Kishi Y., Littlefield B.A., Yu M.J. (2000). Structure–activity relationships of halichondrin B analogues: Modifications at C. 30–C. 38. Bioorg. Med. Chem. Lett..

[81] Nicolaou K., Pan S., Shelke Y., Das D., Ye Q., Lu Y., Sau S., Bao R., Rigol S. (2021). A reverse approach to the total synthesis of halichondrin B. J. Am. Chem. Soc..

[82] Yoshimura T., Yakushiji F., Kondo S., Wu X., Shindo M., Shishido K. (2006). Total synthesis of (+)-lasonolide A. Org. Lett..

[83] Trost B.M., Stivala C.E., Hull K.L., Huang A., Fandrick D.R. (2014). A concise synthesis of (−)-lasonolide A. J. Am. Chem. Soc..

[84] Kang S.H., Kang S.Y., Choi H.-W., Kim C.M., Jun H.-S., Youn J.-H. (2004). Stereoselective total synthesis of the natural (+)-lasonolide A. Synthesis.

[85] Ghosh A.K., Gong G. (2008). Total Synthesis of Potent Antitumor Agent (−)-Lasonolide A: A Cycloaddition-Based Strategy. Chem. Asian J..

[86] Song H.Y., Joo J.M., Kang J.W., Kim D.-S., Jung C.-K., Kwak H.S., Park J.H., Lee E., Hong C.Y., Jeong S. (2003). Lasonolide A: Structural revision and total synthesis. J. Org. Chem..

[87] Ghosh A.K., Gong G. (2007). Enantioselective total synthesis of macrolide antitumor agent (−)-lasonolide A. Org. Lett..

[88] Kang S.H., Kang S.Y., Kim C.M., Choi H.-W., Jun H.-S., Lee B.M., Park C.M., Jeong J.W. (2003). Total synthesis of natural (+)-lasonolide A. Angew. Chem..

[89] Trost B.M., Stivala C.E., Fandrick D.R., Hull K.L., Huang A., Poock C., Kalkofen R. (2016). Total synthesis of (−)-lasonolide A. J. Am. Chem. Soc..

[90] Trost B.M., Cregg J.J. (2015). Ruthenium-catalyzed alkene–alkyne coupling of disubstituted olefins: Application to the stereoselective synthesis of trisubstituted enecarbamates. J. Am. Chem. Soc..

[91] Pettit G.R., Kamano Y., Fujii Y., Herald C.L., Inoue M., Brown P., Gust D., Kitahara K., Schmidt J.M., Doubek D.L. (1981). Marine animal biosynthetic constituents for cancer chemotherapy. J. Nat. Prod..

[92] Pettit G.R., Kamano Y., Dufresne C., Cerny R.L., Herald C.L., Schmidt J.M. (1989). Isolation and structure of the cytostatic linear depsipeptide dolastatin 15. J. Org. Chem..

[93] Pettit G.R., Smith T.H., Arce P.M., Flahive E.J., Anderson C.R., Chapuis J.-C., Xu J.-P., Groy T.L., Belcher P.E., Macdonald C.B. (2015). Antineoplastic agents. 599. Total synthesis of dolastatin 16. J. Nat. Prod..

[94] Pika J., Faulkner D.J. (1995). A reinvestigation of the didemnaketals from the Palauan ascidian *Didemnum* sp. Nat. Prod. Lett..

[95] Potts B.C., Faulkner D.J., Chan J.A., Simolike G.C., Offen P., Hemling M.E., Francis T.A. (1991). Didemnaketals A and B, HIV-1 protease inhibitors from the ascidian *Didemnum* sp. J. Am. Chem. Soc..

[96] Nicolaou K., Bella M., Chen D.Y.K., Huang X., Ling T., Snyder S.A. (2002). Total synthesis of diazonamide A. Angew. Chem..

[97] Nicolaou K., Bheema Rao P., Hao J., Reddy M.V., Rassias G., Huang X., Chen D.Y.K., Snyder S.A. (2003). The second total synthesis of diazonamide A. Angew. Chem. Int. Ed..

[98] Knowles R.R., Carpenter J., Blakey S.B., Kayano A., Mangion I.K., Sinz C.J., MacMillan D.W. (2010). Total synthesis of diazonamide A. Chem. Sci..

[99] Saxena S. (2015). Applied Microbiology.

[100] Erba E., Bergamaschi D., Ronzoni S., Faretta M., Taverna S., Bonfanti M., Catapano C., Faircloth G., Jimeno J., D’incalci M. (1999). Mode of action of thiocoraline, a natural marine compound with anti-tumour activity. Br. J. Cancer.

[101] Dahiya R., Dahiya S., Fuloria N.K., Jankie S., Agarwal A., Davis V., Sahadeo V., Radhay V., Ramsubhag Y., Mullings W. (2021). Natural Thiazoline-based cyclodepsipeptides from marine cyanobacteria: Chemistry, bioefficiency and clinical aspects. Curr. Med. Chem..

[102] Boger D.L., Ichikawa S. (2000). Total syntheses of thiocoraline and BE-22179: Establishment of relative and absolute stereochemistry. J. Am. Chem. Soc..

[103] Dahiya R., Dahiya S., Kumar P., Kumar R.V., Dahiya S., Kumar S., Saharan R., Basu P., Mitra A., Sharma A. (2021). Structural and biological aspects of natural bridged macrobicyclic peptides from marine resources. Arch. Pharm..

[104] Edler M.C., Fernandez A.M., Lassota P., Ireland C.M., Barrows L.R. (2002). Inhibition of tubulin polymerization by vitilevuamide, a bicyclic marine peptide, at a site distinct from colchicine, the vinca alkaloids, and dolastatin 10. Biochem. Pharmacol..

[105] Wipf P., Reeves J.T., Day B.W. (2004). Chemistry and biology of curacin A. Curr. Pharm. Des..

[106] Rinehart K., Fregeau N., Warwick R., Garcia Gravalos D., Avila J., Faircloth G. (1999). Spisulosine Compounds Having Antitumor Activity.

[107] Ganesher A., Chaturvedi P., Sahai R., Meena S., Mitra K., Datta D., Panda G. (2020). New Spisulosine Derivative promotes robust autophagic response to cancer cells. Eur. J. Med. Chem..

[108] Gonda J., Fazekašová S., Martinková M., Mitríková T., Roman D., Pilátová M.B. (2019). Synthesis and biological activity of sphingosines with integrated azobenzene switches. Org. Biomol. Chem..

[109] Warabi K., Matsunaga S., van Soest R.W.M., Fusetani N. (2003). Dictyodendrins A−E, the First Telomerase-Inhibitory Marine Natural Products from the Sponge Dictyodendrilla v erongiformis. J. Org. Chem..

[110] Gowan S.M., Harrison J.R., Patterson L., Valenti M., Read M.A., Neidle S., Kelland L.R. (2002). A G-quadruplex-interactive potent small-molecule inhibitor of telomerase exhibiting in vitro and in vivo antitumor activity. Mol. Pharmacol..

[111] Fürstner A., Domostoj M.M., Scheiper B. (2005). Total synthesis of dictyodendrin B. J. Am. Chem. Soc..

[112] Fürstner A., Domostoj M.M., Scheiper B. (2006). Total syntheses of the telomerase inhibitors dictyodendrin B, C, and E. J. Am. Chem. Soc..

[113] Buchgraber P., Domostoj M.M., Scheiper B., Wirtz C., Mynott R., Rust J., Fürstner A. (2009). Synthesis-driven mapping of the dictyodendrin alkaloids. Tetrahedron.

[114] Okano K., Fujiwara H., Noji T., Fukuyama T., Tokuyama H. (2010). Total synthesis of dictyodendrin A and B. Angew. Chem. Int. Ed..

[115] Tokuyama H. (2021). Construction of N-heterocycles fused with a highly substituted benzene ring by a benzyne-mediated cyclization/functionalization cascade reaction and its application to the total synthesis of marine natural products. Chem. Pharm. Bull..

[116] Hirao S., Yoshinaga Y., Iwao M., Ishibashi F. (2010). A formal total synthesis of the telomerase inhibitor dictyodendrin B. Tetrahedron Lett..

[117] Ayats C., Soley R., Albericio F., Alvarez M. (2009). Synthesis of the pyrrolo [2,3-c] carbazole core of the dictyodendrins. Org. Biomol. Chem..

[118] Hirao S., Sugiyama Y., Iwao M., Ishibashi F. (2009). Synthetic approach to telomerase inhibitor dictyodendrin B: Synthesis of the pyrrolo [2,3-c] carbazole core. Biosci. Biotechnol. Biochem..

[119] Liang J., Hu W., Tao P., Jia Y. (2013). Total synthesis of dictyodendrins B and E. J. Org. Chem..

[120] Ghosal P., Shaw A.K. (2010). An efficient total synthesis of the anticancer agent (+)-spisulosine (ES-285) from Garner’s aldehyde. Tetrahedron Lett..

[121] Amarante G.W., Cavallaro M., Coelho F. (2010). Highly diastereoselective total synthesis of the anti-tumoral agent (±)-Spisulosine (ES285) from a Morita–Baylis–Hillman adduct. Tetrahedron Lett..

[122] Dinda S.K., Das S.K., Panda G. (2010). Asymmetric total syntheses of spisulosine, its diastereo-and regio-isomers. Tetrahedron.

[123] Fabišíková M., Martinková M., Hirková S., Gonda J., Pilátová M.B., Gönciová G. (2016). Total synthesis and the anticancer activity of (+)-spisulosine. Carbohydr. Res..

[124] Abushanab E., Vemishetti P., Leiby R.W., Singh H.K., Mikkilineni A.B., Wu D.C., Saibaba R., Panzica R.P. (1988). The chemistry of L-ascorbic and D-isoascorbic acids. 1. The preparation of chiral butanetriols and-tetrols. J. Org. Chem..

[125] Pitts A.K., O’Hara F., Snell R.H., Gaunt M.J. (2015). A Concise and Scalable Strategy for the Total Synthesis of Dictyodendrin B Based on Sequential C- H Functionalization. Angew. Chem. Int. Ed..

[126] Matsuoka J., Inuki S., Matsuda Y., Miyamoto Y., Otani M., Oka M., Oishi S., Ohno H. (2020). Total Synthesis of Dictyodendrins A–F by the Gold-Catalyzed Cascade Cyclization of Conjugated Diyne with Pyrrole. Chem. Eur. J..

